# Artificial intelligence and its impact on the domains of universal health coverage, health emergencies and health promotion: An overview of systematic reviews

**DOI:** 10.1016/j.ijmedinf.2022.104855

**Published:** 2022-10

**Authors:** Antonio Martinez-Millana, Aida Saez-Saez, Roberto Tornero-Costa, Natasha Azzopardi-Muscat, Vicente Traver, David Novillo-Ortiz

**Affiliations:** a Instituto Universitario de Investigación de Aplicaciones de las Tecnologías de la Información y de las Comunicaciones Avanzadas (ITACA), Universitat Politècnica de València, Camino de Vera S/N, Valencia 46022, Spain; b Division of Country Health Policies and Systems, World Health Organization, Regional Office for Europe, Copenhagen, Denmark

**Keywords:** Machine learning, Universal health coverage, Health emergencies, Health and well-being, European region, AI, Artificial intelligence, BHW, Better Health and Well-being, DL, Deep Learning, EPW, European Programme of Work, HEP, Health Emergencies Protection, ML, Machine Learning, NL, Neural Learning, RL, Reinforcement Learning, SVM, Support Vector Machines, GPW13, The Thirteenth General Programme of Work, UHC, Universal Health coverage, WHO, World Health Organization

## Abstract

**Background:**

Artificial intelligence is fueling a new revolution in medicine and in the healthcare sector. Despite the growing evidence on the benefits of artificial intelligence there are several aspects that limit the measure of its impact in people’s health. It is necessary to assess the current status on the application of AI towards the improvement of people’s health in the domains defined by WHO’s Thirteenth General Programme of Work (GPW13) and the European Programme of Work (EPW), to inform about trends, gaps, opportunities, and challenges.

**Objective:**

To perform a systematic overview of systematic reviews on the application of artificial intelligence in the people’s health domains as defined in the GPW13 and provide a comprehensive and updated map on the application specialties of artificial intelligence in terms of methodologies, algorithms, data sources, outcomes, predictors, performance, and methodological quality.

**Methods:**

A systematic search in MEDLINE, EMBASE, Cochrane and IEEEXplore was conducted between January 2015 and June 2021 to collect systematic reviews using a combination of keywords related to the domains of universal health coverage, health emergencies protection, and better health and wellbeing as defined by the WHO’s PGW13 and EPW. Eligibility criteria was based on methodological quality and the inclusion of practical implementation of artificial intelligence. Records were classified and labeled using ICD-11 categories into the domains of the GPW13. Descriptors related to the area of implementation, type of modeling, data entities, outcomes and implementation on care delivery were extracted using a structured form and methodological aspects of the included reviews studies was assessed using the AMSTAR checklist.

**Results:**

The search strategy resulted in the screening of 815 systematic reviews from which 203 were assessed for eligibility and 129 were included in the review. The most predominant domain for artificial intelligence applications was Universal Health Coverage (N = 98) followed by Health Emergencies (N = 16) and Better Health and Wellbeing (N = 15). Neoplasms area on Universal Health Coverage was the disease area featuring most of the applications (21.7 %, N = 28). The reviews featured analytics primarily over both public and private data sources (67.44 %, N = 87). The most used type of data was medical imaging (31.8 %, N = 41) and predictors based on regions of interest and clinical data. The most prominent subdomain of Artificial Intelligence was Machine Learning (43.4 %, N = 56), in which Support Vector Machine method was predominant (20.9 %, N = 27). Regarding the purpose, the application of Artificial Intelligence I is focused on the prediction of the diseases (36.4 %, N = 47). With respect to the validation, more than a half of the reviews (54.3 %, N = 70) did not report a validation procedure and, whenever available, the main performance indicator was the accuracy (28.7 %, N = 37). According to the methodological quality assessment, a third of the reviews (34.9 %, N = 45) implemented methods for analysis the risk of bias and the overall AMSTAR score below was 5 (4.01 ± 1.93) on all the included systematic reviews.

**Conclusion:**

Artificial intelligence is being used for disease modelling, diagnose, classification and prediction in the three domains of GPW13. However, the evidence is often limited to laboratory and the level of adoption is largely unbalanced between ICD-11 categoriesand diseases. Data availability is a determinant factor on the developmental stage of artificial intelligence applications. Most of the reviewed studies show a poor methodological quality and are at high risk of bias, which limits the reproducibility of the results and the reliability of translating these applications to real clinical scenarios. The analyzed papers show results only in laboratory and testing scenarios and not in clinical trials nor case studies, limiting the supporting evidence to transfer artificial intelligence to actual care delivery.

## Highlights

• An overview of systematic reviews on the application of AI including 129 studies.
• AI use is prominent in Universal Health Coverage, featuring image analysis in neoplasms.
• Half of the reviews did not evaluate validation procedures nor reporting guidelines.
• Risk of bias was only included un a third of the reviews.
• There is not sufficient evidence to transfer AI to actual healthcare delivery.

## 1 Introduction

The Thirteenth General Programme of Work (GPW 13) defines the World Health Organization’s (WHO) strategy for the period between 2019 and 2023 which focuses on measurable impacts on people’s health [1]. Based on the GPW 13, the core priorities and the roadmap for the 53 countries of the WHO European region are described in the European Programme of Work 2020–2025 (EPW) [2]. The GPW 13 and the EPW aim to transform public health, focusing on measurable impacts on people’s health at the national level with three core features: enhanced Universal Health coverage (UHC), Health Emergencies Protection (HEP), and Better Health and Well-being (BHW).

The UHC domain involves primary care, community care and person-centered health systems [3]. Health promotion and disease prevention are the key principles in which UHC should be constructed and maintained. Furthermore, primary care services should also ensure access to curative, rehabilitative and palliative health services regardless the geographical location and the financial status [4]. The HEP domain is focused on preparing health care systems to better react when a health emergency is declared, this is to provide adequate and timely services that range from disease prevention to life-saving interventions. The BHW domain is focused on improving the general health and wellbeing of people, involving the prevention of noncommunicable disease, promoting mental health, minimizing and eradicating high impact communicable disease and addressing the health effects of climate change.

Artificial intelligence (AI) is a discipline which seeks to reproduce human-like ways of perceiving, reasoning, learning, and solving problems and is an area of interest in clinical applications [5]. AI expands traditional statistical techniques, allowing to extract information to support decision-making and research. These methods have been deployed in many clinical research areas and technological domains [6], [7], [8].

AI has been implemented in healthcare over a wide typology of clinical applications, for example, from molecular and genetic testing to medical images of different modalities, diagnostic codes and social media [9]. The ultimate goal of AI is to learn and identify associations between data and outcomes of interest [10]. AI needs data generated from healthcare activities such as diagnosis, treatments and follow-up to develop, test and validate algorithms. Digitalized data in healthcare is available in a wide range of formats, including structured and non-structured schemas [11].

The landscape of AI methods can be divided in four main categories: regression and probabilistic methods, machine learning, deep learning and reinforcement learning [12], [13]. One of the most prominent areas of AI is Machine Learning (ML), in which models can adapt to improve their performance according to the changes in the data and the experiences [14]. ML algorithms combine the strengths of computer science and data science to find the optimal fit between theoretical approaches and data-driven solutions, enabling the development of tools that can solve problems human cannot do in reasonable timespans. The basic principle relies in the ability of the model to predict the output whenever new input data is given and thus inform about possible scenarios to understand the information. There are two major approaches in ML, the supervised learning to solve problems of classification and regression based on sets of labelled input data and the unsupervised learning, which seeks to find patterns in sets of unexplained and unlabeled data.

Linear and logistic regression, Support Vector Machines (SVM) and decision trees are relevant techniques in the supervised approach. An extended use of unsupervised learning focuses on finding associations of data in clusters and the identification of principal components that explain multidimensional data [15]. These associations do not consider the outcome information but the nature of the input data, providing categories of patients based on their similarities. Typical algorithms for clustering are k-means and hierarchical clustering. The regular pipeline involves using unsupervised learning to pre-process data and select features that explain the nature of data, to thereafter apply supervised learning to provide clinically relevant results. These modelling techniques pursue to minimize the classification (or misclassification) error (e.g.: the quadratic loss function).

The evolution of ML is known as Neural Learning (NL) and has the ability of generating multiple non-linear combinations of data for creating exhaustive models. This approach combines artificial neural networks that replicate the structure and behavior of a human brain in the way it connects several processing units in multiple layers to adjust their configuration based on the data. Artificial neurons are grouped into layers driving signals from the input to the output. The combination of both ML and NL has led to the definition of Deep Learning (DL), which consists in a hierarchical combination of processing units grouped and connected into layers to transform and extract information [16]. DL approach conducted to the definition of Reinforcement Learning (RL) in which the algorithms learn actions based on the maximization of a predefined reward [17]. The basic principle of RL is based on the interaction of an agent that makes decisions and its environment with the goal of reaching preferable states. After every interaction the agent receives feedback, which can be positive (a reinforcement) or negative, and then RL model will prosecute the actions that maximizes the number of positive rewards.

AI involves a wide variety of methods that expand traditional statistical techniques and can find patterns that support the process of decision making as well as the formulation of hypotheses in the domains of UCH, EHP and BHW. AI can provide powerful tools to automate tasks and to support and inform clinicians, epidemiologists and policy makers on what are the most efficient strategies to promote health at a population and individual level. But, due to the broad range of applications of AI in healthcare is necessary to assess the current status on the application of AI and in which way they can improve people’s health. This overview of systematic reviews has the objective of providing a comprehensive landscape on the most recent evidence on the application of AI to in the three domains defined by the GPW 13 and the EPW. The overview includes regression algorithms, machine learning, deep learning and reinforcement learning approaches, and their application in any medical and clinical specialty domain. The selection criteria are defined as real AI applications on health and care services that can be transferred to real clinical scenarios. The ultimate goal is to provide a comprehensive and updated map on the fields of application of AI to improve people’s health and reveal the medical prominent specialties, the modelling techniques, what type of data is used and, importantly, the methodological quality of the recent scientific literature.

## 2 Methods

### 2.1 Search strategy

**Fig. 1:**
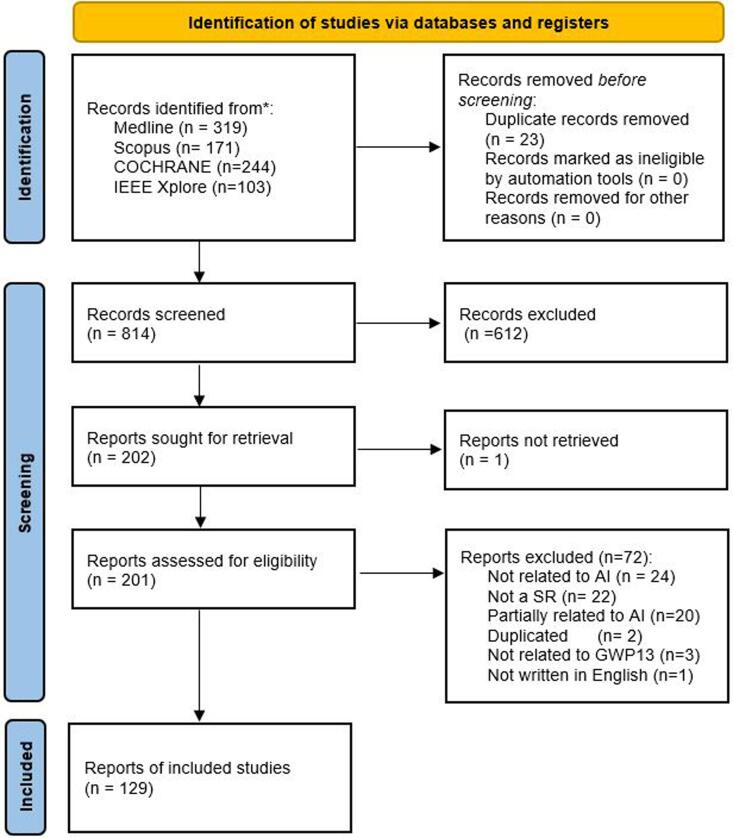
Systematic Reviews selection process.

A systematic literature search on systematic reviews featuring qualitative and/or meta-analysis was conducted using four electronic databases: MEDLINE, IEEE Xplore, EMBASE and COCHRANE. The search string included a combination of keywords (artificial intelligence OR machine learning OR deep learning) AND (universal health coverage OR emergency care OR public health) and the search queries listed in Appendix I. The Preferred Reporting Items for Systematic reviews and meta-analyses (PRISMA 2020 statement) [18] was used to funnel the article selection process (Fig. 1). From the total articles (n = 837), duplicated entries were removed (n = 23). No additional studies were excluded using automation tools or because of other reasons. Afterwards, the hits were screened based on the title and abstract to identify relevant studies matching three basic criteria: 1) Being a Systematic Review; 2) Analysis of Artificial Intelligence modelling; 3) Related to one domain of the GPW 13 and the EPW. Once the relevant articles were identified (n = 203), a full-text review was performed to assess their eligibility. Four articles were excluded because of the following reasons: not in English (n = 1), duplicated (n = 2) and not retrieved – it was not possible to find the full publication available – (n = 1). A total of 72 articles were excluded after the in-depth assessment because the entry was not related or partially related to AI (n = 24) (the paper is not focused on AI development or validation), it was not a systematic review (n = 21), they were partially related to AI (n = 20), duplicated studies (n = 2), not related to the GPW 13 and EPW domains (n = 3) and not written in English (n = 1). Two authors participated in the abstract screening and full- text review. When authors did not reach a consensus a third author evaluated the possible exclusion to break the deadlock.

### 2.2 Eligibility criteria

Systematic reviews were included in the overview if they reported on the implementation and evaluation of artificial intelligence in a health or disease area related to universal health coverage, emergencies and better health and wellbeing. Systematic reviews covering a specific medical technology (e.g.: radiology) were included in the analysis. Studies on data-driven models, natural language processing and image processing were included only if they explicitly designated a relationship with a disease or a health management area. Editorials, protocols and reports were excluded. Also, studies not following the methodology of a systematic review were excluded.

### 2.3 Data extraction

The following data were extracted systematically for all the studies: authors, title, year, journal, GPW 13 and EPW domain, relevant type of disease according to the International Code of Disease, version eleven (ICD-11), number of included studies, type of data source (public/private), data types used by the models, predictors used by the models (input data categories), outcome of the model (screening, diagnose, classification, treatment), type of modelling (regression based, machine learning, deep learning or reinforcement learning), predominant modelling technique, validation methodology (clinical trial, case study or statistical appraisal), performance indicators and risk of bias assessment. Two authors participated in the data extraction and classification of the articles. When authors did not reach a consensus a third author evaluated the entry and assigned the correspondent label.

### 2.4 Quality assessment

Included studies were evaluated with the Assessment of Multiple Systematic Reviews checklist (AMSTAR) [19] to assess the methodological quality of the systematic review. Every item of the AMSTAR checklist was scored with 1 point if it was successfully reported and 0 points otherwise.

## 3 Results

The analysis of the systematic reviews was divided into the three domains of the GPW 13 and the EPW priorities, that is 98 in universal health coverage, 16 in health emergencies protection and 15 in better health and wellbeing. Appendix II contains the complete list of the systematic reviews included in the overview, their domain and related ICD-11 chapter and the number of included studies (qualitative and meta-analysis). Appendix III presents a descriptive analysis of the systematic review with respect to the health/disease area, the type of data and modelling technique and the highlight challenges and opportunities. Appendix IV depicts the descriptive analysis for each GPW13- EPW domain of the extracted data (quantitative and qualitative). Appendix V contains the AMSTAR evaluation scores and the individual quality of each review included in the analysis.

With respect to the domain of universal health coverage, neoplasms are the predominant disease application (N = 28), followed by mental and behavioral disorders (N = 17), diseases of the circulatory system (N = 9) and the musculoskeletal system (N = 8). In the domain of emergencies, the predominant disease category is infectious or parasitic diseases (N = 13) and in the domain of better health and wellbeing all fall into the category of factors influencing health status or contact with health services (N = 14). The studies included heterogeneous designs, focusing on different approaches to the health promotion and the disease management, sources of data, target population, predictors and assessment of the outcomes. The following subsections describe the collated results of the review for each of the GPW 13 and the EPW domains.

### 3.1 Artificial intelligence for areas related to universal health coverage

**Fig. 2:**
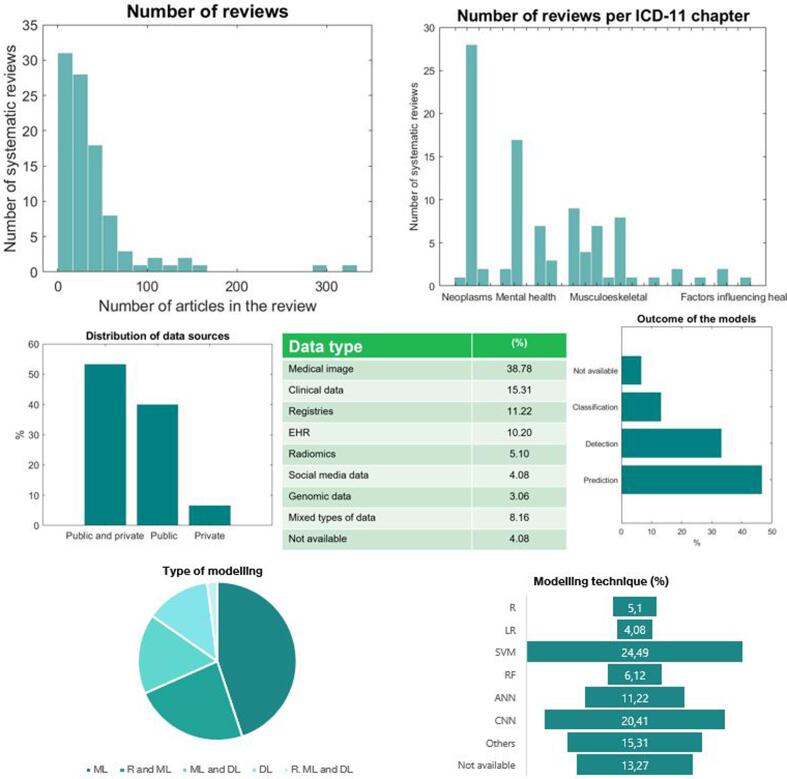
Dashboard and descriptive analytics on the use of AI in areas related to Universal Health Coverage.

The application of AI in UHC domain is mainly focused on neoplasms (N = 28) [20], [21], [22], [23], [24], [25], [26], [27], [28], [29], [30], [31], [32], [33], [34], [35], [36], [37], [38], [39], [40], [41], [42], [43], [44], [45], [46], [47] and secondary on mental health (N = 17) [48], [49], [50], [51], [52], [53], [54], [55], [56], [57], [58], [59], [60], [61], [62], [63], [64] as depicted in Fig. 2. There is an intermediate group including diseases of the circulatory system (N = 9) [65], [66], [67], [68], [69], [70], [71], [72], [73], diseases of the musculoskeletal system (N = 8) [74], [75], [76], [77], [78], [79], [80], [81], diseases of the digestive system (N = 7) [82], [83], [84], [85], [86], [87], [88] and the nervous system (N = 7) [89], [90], [91], [92], [93], [94], [95]. There are a few reviews focused on other categories such as diseases of respiratory system (N = 4) [96], [97], [98], [99], visual system (N = 3) [100], [101], [102] and the other chapters include only one or two systematic reviews [103], [104], [105], [106], [107], [108], [109], [110], [111], [112], [113], [114], [115], [116]. The number of studies included in the reviews is 41.29 ± 41.9 (mean ± standard deviation) with an IQR = [16–47]. In the predominant categories, the number of reviews is sparse with 34.23 ± 31.78 for neoplasms, 43.00 ± 36.97 for factors influencing health and 57.93 ± 73.29 for mental health. Only the 20 of these reviews included a *meta*-analysis, 5 of them belonged to the disease area of neoplasms while the other categories included only one or two meta-analysis.

The studies included in the reviews featured analytics primarily over public and private data sources (68.37 %) and less on public data (28.57 %). Only a 3.06 % did not disclose the sources of data. Medical imaging (38.78 %) is the most used type of data, followed by clinical data from laboratory tests and examinations (15.31 %), registries from clinical studies (11.22 %) and electronic health records (10.20 %). Some reviews used mixed types of data, but these account for the 8.16 % of them. Other reviews were focused on social media data (4.08 %) and genomic data (3.06 %). With respect to the predictors (the specific type of variables used develop the models), the majority is based on Regions of Interest (39.8 %) and mixed predictors such as imaging features, clinical assessment and notes (15.3 %). The reviews analyzed similarly clinical and demographic variables (6.12 %) and signal features and vital signs (6.12 %). A few reviews were focused on the analysis of notes (5.1 %) and histological data (4.1 %). Importantly, less than a quarter of the reviews did not disclose the type of predictors used by the models (23.5 %).

In the domain of UHC the application of AI is focused on the prediction of the diseases (32.65 %) and the detection (24.49 %). Some reviews focused on the classification of diseases degrees and severity scales (18.37 %). A few reviews focused on segmentation (4.08 %) and other unspecific outcomes (6.12 %). Machine learning techniques accounted for the 44.9 % of the types of AI, and their combination with regression methods (23.47 %) and deep learning techniques (16.33 %). Deep learning was used in the 13.27 % of the reviews. The predominant modelling technique was Support Vector Machines (24.49 %) and Convolutional Neural Networks (20.41 %).

With respect to the validation, slightly more than a half (52.04 %) did not report any validation procedure. Almost a third of the reviews reported internal validation (32.65 %), only 5.1 % reported external validation, and 12.24 % reported both internal and external validation. When described, the main performance indicator was de performance (31.63 %), followed by a compendium of indicators of C statistic (AUC), sensitivity and specificity (24.49). The AUC was reported as the single indicator in the 20.41 % of the reviews. and the sensitivity and specificity on the 12.24 %,

Regarding the quality assessment, the 61.22 % of the reviews did not implement any method for analyzing the risk of bias, and for the remaining 38.78 % of reviews performing it, QUADAS-2 was the most used method (17.35 %). From the 71 qualitative analysis, 19 (26.7 %) performed a quality assessment, and from the 27 meta-analysis (some studies included a qualitative and a meta-analysis), 20 (74.1 %) performed a quality assessment. The overall AMSTAR score was low (4.05 ± 1.99), with a few reviews yielding scores over 5 points. Table 1 in the Appendix III contains the complete descriptive analysis of the systematic reviews classified into areas related to Universal Health Coverage domain.

### 3.2 Artificial intelligence for areas related to health emergencies protection

**Fig. 3:**
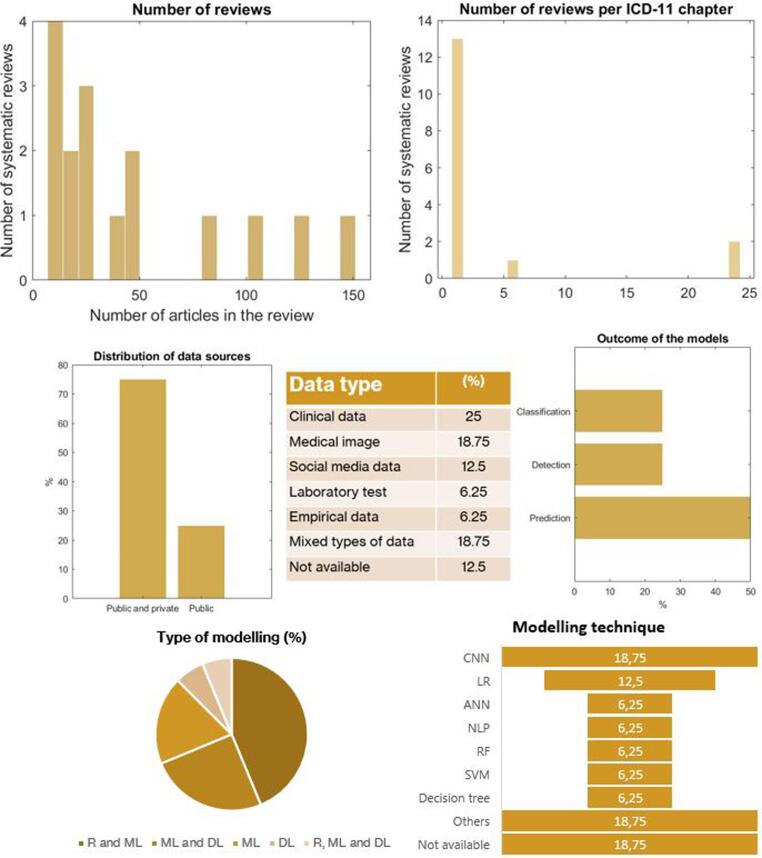
Dashboard and descriptive analytics on the use of AI in areas related to Health Emergencies Protection.

The application of AI in HEP is mainly focused on infectious or parasitic diseases (N = 13) [117], [118], [119], [120], [121], [122], [123], [124], [125], [126], [127], [128] and secondary on factors influencing health (N = 2) [129], [130] and mental health (N = 1) [131], as depicted in Fig. 3. SARS-CoV-2 was the main topic on 10 of these reviews. The number of studies included in the reviews is 46.75 ± 44.66 with an IQR = [16–65.5]. In the predominant category, the number of reviews is 52.46 ± 47.98. Only the 12.5 % of these reviews include meta-analysis, one of them belonged to infectious diseases and the other according to factors influencing health.

The studies included in the reviews featured primarily over public and private data sources (75 %) and less on public data (25 %). In this case, the most used typed of data is clinical data (25 %), followed by medical imaging (18.75 %), social media data (12.5 %), laboratory test (6.25 %) and empirical data (6.25 %). Besides, some reviews used mixed types of data (18.75 %). With respect to the predictors, the majority is based on regions of interest (25 %), followed by analysis of notes (12.5 %), clinical or demographic variables (12.5 %), genome sequences (6.25 %) and mixed predictors (6.25 %). Nevertheless, 37.5 % of the reviews did not disclose the type of predictors used by the models.

On the other hand, the application of AI in the domain of Health Emergencies Protection is focused on the prediction of the diseases (50 %), followed by the detection (25 %) and classification of diseases degrees and severity scales (25 %). Machine learning and regression techniques accounted for the 43.75 % of the types of AI, 25 % of machine learning and deep learning methods and 18.75 % of only machine learning. The predominant modelling technique was Convolutional Neural Networks (18.75 %) and Logistic regression (12.5 %).

With respect to the validation, more than a half (56.25 %) did not report any validation procedure. A quarter of the reviews (25 %) reported both internal and external validation, and 18.75 % reported only internal validation. No external validation was reported. In addition, the main performance indicator was the accuracy (25 %) and AUC (25 %), followed by a compendium of indicators (12.5 %) such as sensitivity and specificity, C statistic and R^2^.

Regarding the quality assessment, the 75 % of the reviews did not implement any method for analyzing the risk of bias. From the 14 qualitative analysis, 3 (21.4 %) performed a quality assessment, and from the 2 *meta*-analysis, only one (50 %) performed a quality assessment. The PROBAST tool was the most used method (17.35 %). The overall AMSTAR score was low (4.06 ± 1.98), with a few reviews yielding scores over 5 points. Table 2 in the Appendix III contains the complete descriptive analysis of the systematic reviews classified into health emergencies protection.

### 3.3 Artificial intelligence for areas related to a better health and Well-being

**Fig. 4:**
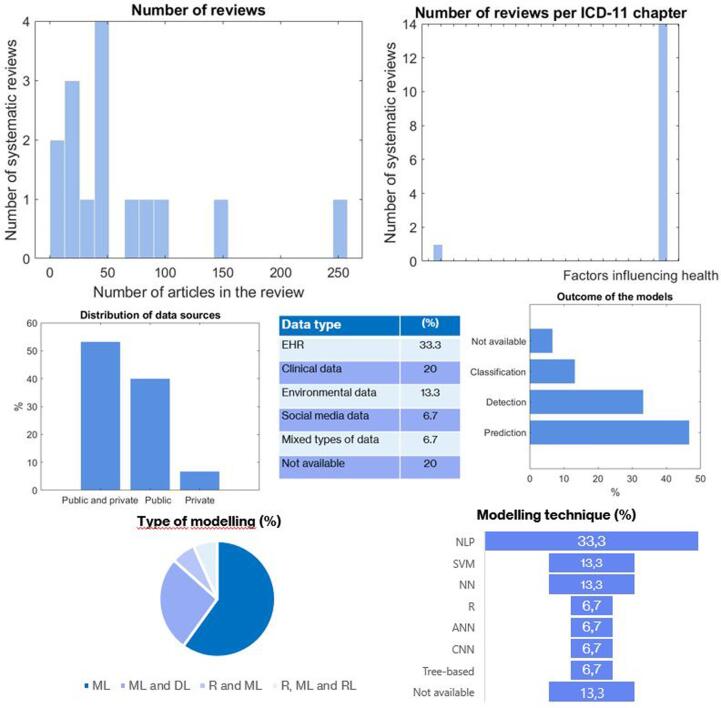
Dashboard and descriptive analytics on the use of AI in areas related to a Better Health and Wellbeing.

The application of AI in BHW domain is mainly focused on factors influencing health (N = 14) [132], [133], [134], [135], [136], [137], [138], [139], [140], [141], [142], [143], [144], [145] and secondary on mental health (N = 1) [146], as depicted in Fig. 4. The number of studies included in the reviews is 62 ± 66.36 with an IQR = [18–81.25]. In the predominant category, the number of reviews is 65.21 ± 67.65. No review included *meta*-analysis.

The studies included in the reviews featured analytics primarily over public and private data sources (53.3), followed by public data (40 %) and less on private data (6.7 %). Electronic health record (33.3) is the most used typed of data, followed by clinical data (20 %) and environmental data (13.3 %). Other reviews were focused on social media data (6.7 %) and mixed types of data (6.7 %). According to predictors, the majority is based on analysis of notes (40 %), followed by clinical variables (6.7 %) and mixed predictors (6.7 %). Importantly, almost the half (46.7 %) of the reviews did not disclose the type of predictors used by the models.

In the domain of Better health and Wellbeing, the application of AI is focused on the prediction of the diseases (46.7 %), secondary on detection (33.3 %) and classification of diseases degrees and severity scales (13.3 %). Machine learning techniques accounted the 60 % of the types of AI and their combination with deep learning (26.7 %). The predominant modelling technique was Natural Language Processing (NLP) (33.3 %), followed by Support Vector Machine (13.3 %) and Neural Networks (13.3 %).

Regarding the validation techniques, more than a half (66.7 %) did not report any validation procedure. A quarter of the reviews (26.7 %) reported internal validation and only 6.7 % reported both internal and external validation. The main performance indicator was a compendium of indicators (20 %) such as AUC, sensitivity and specificity, followed by the accuracy (13.3 %).

When it comes to the quality assessment, the 80 % of the reviews did not implement any method for analysis the risk of bias. None of the included studies in BHW performed a meta-analysis. Cochrane’s tool was the most used method (13.3 %). The overall AMSTAR score was low (3.67 ± 1.54), with a few reviews yielding scores over 5 points. Table 1 in the Appendix III contains the complete descriptive analysis of the systematic reviews classified into BHW.

## 4 Discussion

Despite the recent increase in the AI literature and the publication of systematic reviews about AI applications in healthcare, the research in this field is focused on certain diseases. The type of data which algorithms are implemented with and the availability in public repositories could be a possible cause. Neoplasms is one of the most prominent areas due to the use of different medical image modalities and the recent advances in image processing techniques with DL approaches, moreover, because an accurate and on-time diagnose is crucial to determine the treatment and minimize the causes that lead to the death in neoplasms. One of the most recurrent applications of AI is the early diagnosis (predictions) and classification of disease severity, which can be improved by using other data sources such as Electronic Medical Reports. The impact of AI in care delivery in the chosen domains cannot be measured as all the reviewed studies featured results in laboratory settings and did not include clinical evaluation.

The effective management of public health systems is a multifactorial responsibility with a wide range of actors and effects. Like any other medical act, the provision of care services at a populational level involves the screening and diagnosis of certain conditions, the treatment and the follow-up of those conditions [71], [95], [115]. In this context, the use of AI has demonstrated that it can provide powerful tools to support and inform decisions and even automate tasks to aid clinicians, epidemiologists and policy makers on the most efficient strategies to promote health at a population level [137], including the current COVID-19 pandemic [125]. However, despite the great advances and the high level of maturity of AI in certain clinical domains, the review of systematic reviews leads to conclude that the use of AI is still scarce in clinical practice and depends strongly upon the clinical application domain [58], [72]. Published evidence mainly consists of tests in laboratory settings and early-phase validation of ML and DL models.

This overview is a broad summary on the development and application status of AI in healthcare but also stands as an assessment on the quality of the systematic reviews in the use of AI. The use of reporting guidelines on the methodology related to the elaboration and selection of papers for the systematic review is a good practice implemented in the vast majority of the reviews. However, the quality assessment of individual studies is only implemented in a minority of them. Quality assessment tools and risk of bias methods are an utmost important tool to understand the level of evidence reached by the authors and the real impact on health outcomes. Recently, there have been important updates on standard reporting guidelines such as the CONSORT-AI for clinical trial reports involving AI [147], the SPIRIT-AI for clinical trial protocols involving AI [148], the MI-CLAIM checklist on minimum information about clinical AI modelling [149] and the PROBAST tool to assess the risk of bias and applicability of prediction model studies [150]. These updates and recommendations address important issues related to the development, implementation and evaluation of clinical interventions based on AI in a broad set of factors identified in this overview. Individual studies and systematic reviews should describe the intended workflow in the use of the AI intervention, with a specific statement on the disease context, intended users and the purpose of the intervention.

Despite the rapid growth and generalization of AI in several fields of medicine, this overview shows that there is a limited level of maturity on its use in clinical practice. None of the analyzed reviews reported studies on the real impact of these tools in real clinical scenarios. This outcome shows an important gap between the development of the models in laboratory settings and the implementation of this models under real conditions. However, in some specific clinical areas, the level of maturity of the models is unarguably high and the current body of literature shows consistent results in specific indicators, methodologies, and comparison metrics. These situations should ease the transference of these models to improve the current techniques and protocols used for the assessment of disease conditions.

The type of data used to implement AI models is heterogeneous and frequently not consistent across the same type of clinical domains and applications [151]. Diagnostic medical imaging is the principal data source for Neoplasms, Infections and Surgery among others and constitutes the main trend of research in the application of AI. The analysis of time series and natural language processing from social networks and medical records are emerging fields of research and an interesting field for the development of AI.

### 4.1 Challenges

One of the common pitfalls identified in this overview is the lack of standardization protocol designs on the interventions of AI, including the approaches to perform the statistical analysis of the outcomes. The high level of heterogeneity found in the approaches to select observable variables, outcomes and the performance analysis makes impossible to compare disease-specific cut-off points. The challenge of standardization is not new in clinical research and should be taken under deep consideration when referring to the use of AI in medicine [40]. It is common to find studies which are biased by imbalanced classes, specifically when the study includes limited sample sizes and outcomes that are difficult to measure (e.g.: mental health, suicide, etc.). This affects the interpretation of the results because it does not show the ability of the models to discriminate positive and negative cases. Another common challenge found in the systematic reviews is the heterogeneity of software infrastructures used to collect, store, and analyze personal and clinical data.

One of the most recurrent issues in the analyzed reviews is the scarcity of data and the risk of model overfitting, especially in models developed for medical imaging processing. Penalization techniques are recommended to address overfitting in models, but they should be applied carefully depending on the data size. Penalization techniques could be an unreliable choice when the sample size is small [152].

The use of performance metrics to compare and evaluate AI models is common in the revised systematic reviews. Study design limitations in the statistical analysis can lead to wrong conclusions and under/over-estimations in the accuracy and the classification ability of the models, however, the quality of this models rely on the data used to train and validate them. Beyond issues of data heterogeneity and availability, understanding the data biases applied in the model development is crucial.

Another challenge of AI in clinical practice is that it must show acceptable and reproductible results. Limitations in the reporting of AI models are a challenge to homogenize the use of AI in research and how to adapt the specific models to the particular needs of clinical units and populations in terms of healthcare service characteristics [101], [111]. One of the modelling approaches most frequently used are the decision trees because of its performance and its ability to illustrate in human understandable way how a decision was made. In opposite, other modelling techniques have no ability to indicate how the decision was made without falling into a (frequently) complex mathematical formulation [110].

### 4.2 Opportunities

The overview has also spotted many opportunities in the field of AI and medicine. Future studies should include deeper considerations on the pathophysiology of the specific disease when designing the protocol for the intervention. The incorporation of a control group and using cross-validation will increase the evidence and the credibility on these types of studies [39]. Despite not having found real applications in clinical contexts in which AI drives a decision. Many reviews conclude that ML will play an important role helping clinicians to identify specific indicators and this will lead to a better diagnose, treatment and outcomes.

Open databases and basic principles of data sharing will be paramount to develop and implement AI models, allowing to reproduce results, compare the accuracy of different methods and approaches and confirming scientific findings [153]. The main barrier to modelling lies between laboratory conditions and free-living clinical and practical environments, but still the incorporation of contextual factors will ease the transference of these models to real life scenarios. Digital data interventions have the opportunity to enhance population’s health and wellbeing, health coverage and protection from emergencies, but they should be boosted by the application of ML algorithms in population-based clinical decision making with the use of Big Data and new communication technologies.

The degree of interdisciplinarity of the research teams has demonstrated to be an influential factor in the quality of the research, as demonstrated in the clinical case of thoracic cancer [154]. Studies driven by a clinical meaningful need, supported by an adequate design and focused on a clinical practice target will generate more transferable results. The implementation of sustained educational training programs, such as diplomas, Masters and PhD’s focused at healthcare professionals in collaboration with stakeholders from the engineering fields will allow to build new capacities and teams to spread the theories and findings of AI based tools in medicine [155]. However, their effectiveness and trustworthiness will be only demonstrated through the implementation of well-designed clinical trials, and herein collaborative partnerships can play a significant role for sharing resources, knowledge and scientific expertise between countries to optimize training and research opportunities. An increment in the interdisciplinarity of teams will lead to a more mature and clinical practice-oriented research.

## 5 Conclusion

Artificial intelligence applications in the three domains of GPW13 have proved to increase our insights for disease modelling, diagnose, classification and prediction in a wide range of clinical domains and different scenarios. However, this evidence is often limited to laboratory and testing scenarios. Cross-sectional and longitudinal data from public repositories, clinical registries, clinical trials and other datasets is continuously being used to develop and validate AI models showing excellent results in the context of their respective study designs. However, there is a huge need of improving the methodological reporting of these studies and to improve the robustness of these models to consider variabilities from different sources. Explainable AI is a growing field of research that will respond to the needs of understanding how models are inferred from clinical and health data. The combination of AI modelling and explainable strategies will have a better clinical value in the diagnose and treatment of diseases, allowing healthcare systems to improve the quality of universal healthcare coverage, the responses to emergencies and to support healthier populations.

## Authors’ contributions

AMM, VT, NAM, and DNO designed the study. AMM, ASS, and VT performed first- and second-stage screening, and extracted the presented data. VT solved any disagreements. AMM, ASS and VT carried out the quality assessment. AMM, ASS, RTC, VT, and DNO drafted the manuscript and its final version. All authors contributed to the article and approved the submitted version.

## Disclaimer

DN-O and NA-M are staff members of the WHO. The authors alone are responsible for the views expressed in this article and they do not necessarily represent the decisions, policy, or views of the WHO.

## Declaration of Competing Interest

The authors declare that they have no known competing financial interests or personal relationships that could have appeared to influence the work reported in this paper.
