# Supplementary material for: Artificial intelligence and its impact on the domains of universal health coverage, health emergencies and health promotion: An overview of systematic reviews
Source: Int J Med Inform. 2022 Oct;166:104855. doi: 10.1016/j.ijmedinf.2022.104855 (PMC9551134; doi:10.1016/j.ijmedinf.2022.104855)
Supplement: Supplementary data 1 [file mmc1.docx]

# Appendix I

Combination of queries used to complement the searches in the scientific data bases.

**AI:**

(((("artificial intelligence"[Title/Abstract]) OR ("deep learning"[Title/Abstract])) OR ("machine learning"[Title/Abstract])) OR ("machine intelligence"[Title/Abstract])) OR ("neural networks"[Title/Abstract])

**UHC:**

((((((((((((((((((((((((((((((((((((((((((((((((("universal health coverage") OR (UHC)) OR ("reproductive health")) OR ("maternal health")) OR ("newborn health")) OR ("family planning")) OR ("modern contraceptive")) OR ("postpartum amenorrhoeic")) OR ("child health")) OR ("antenatal care")) OR ("pregnancy")) OR ("child immunization")) OR ("diphtheria-tetanus-pertussis")) OR (DTP3)) OR (MCV2)) OR (PC3V3)) OR ("Infectious disease*")) OR (tuberculosis)) OR (TB)) OR (HIV)) OR ("Human immunodeficiency virus")) OR (malaria)) OR ("water, sanitation AND hygiene")) OR (WASH)) OR ("sanitation")) OR ("hygiene")) OR ("Non-communicable disease*")) OR (NCD*)) OR ("cardiovascular disease*")) OR ("blood pressure")) OR ("hypertension")) OR (diabetes)) OR (tobacco)) OR (smoke)) OR (cancer)) OR ("chronic respiratory disease*")) OR ("chronic obstructive pulmonary disease")) OR (asthma)) OR (hyperglycemia)) OR (hyperlipidemia)) OR ("risk factor*")) OR ("service coverage")) OR ("essential health service*")) OR ("hospital access")) OR ("hospital bed*")) OR ("health worker*")) OR ("health professional*")) OR ("health security")) OR ("international health regulations")) OR (IHR)

**Health emergencies protection:**

(((((((((("emergency prepare") OR ("health emergency")) OR ("emergency prevent")) OR ("vaccine coverage")) OR ("vaccination coverage")) OR ("epidemic")) OR ("pandemic")) OR ("emergency vaccine*")) OR ("notifiable event*")) OR ("emergency detect")) OR ("emergency respond")

**Better Health and Wellbeing:**

((((((((((((((((("childhood stunting") OR ("childhood wasting")) OR ("childhood overweight")) OR (suicide)) OR (alcohol)) OR ("mental health")) OR ("road death*")) OR ("road safety")) OR ("tobacco use")) OR ("partner violence")) OR ("gender violence")) OR ("sanitation service*")) OR ("clean fuel*")) OR ("violence against children")) OR (obesity)) OR (overweight)) OR (diet)) OR ("physical activity")

# Appendix II

List of articles included in the systematic review

| **Columna20** | **DOI** | **GPW13 EPW**  **Domain** | **ICD-11 Chapter** | **Number of included reviews** | **Meta-analysis (Y/N)** |
| --- | --- | --- | --- | --- | --- |
| Salod Z et al, 2020 | 10.4081/jphr.2020.1772 | 1 | 2 | 11 | N |
| Arji G et al, 2019 | 10.1016/j.cmpb.2018.10.017 | 1 | 26 | 42 | N |
| Kothari G et al, 2021 | 10.1016/j.radonc.2020.10.023 | 1 | 2 | 40 | Y |
| Yuan M et al, 2019 | 10.1016/j.jbi.2019.103181 | 3 | 24 | 145 | N |
| M. A. S. A. Husaini et al, 2020 | 10.1109/ACCESS.2020.3038817 | 1 | 2 | Not available | N |
| Bellinger C et al, 2017 | 10.1186/s12889-017-4914-3 | 3 | 24 | 47 | N |
| Kearns WR et al, 2019 | 10.1055/s-0040-1708807 | 3 | 24 | 46 | N |
| N. F. Zulkifli et al, 2020 | 10.1109/ICIMU49871.2020.9243581 | 1 | 6 | 11 | N |
| Wang W et al, 2020 | 10.1371/journal.pone.0234722 | 1 | 8 | 18 | N |
| Gupta V et al, 2020 | 10.3390/s20216100 | 1 | 3 | 27 | N |
| Dreisbach C et al, 2019 | 10.1016/j.ijmedinf.2019.02.008 | 3 | 24 | 21 | N |
| Young IJB et al, 2019 | 10.1016/j.ijmedinf.2019.103971 | 1 | 24 | 35 | N |
| Zhong J et al, 2021 | 10.1007/s00330-020-07221-w | 1 | 2 | 12 | Y |
| Karmegam D et al, 2020 | 10.1017/dmp.2019.40 | 2 | 6 | 18 | N |
| Harris M et al, 2019 | 10.1371/journal.pone.0221339 | 1 | 12 | 53 | N |
| M. N. Islam et al, 2020 | 10.1109/TAI.2021.3062771 | 2 | 1 | 49 | N |
| Lui TKL et al, 2020 | 10.1016/j.gie.2020.06.034 | 1 | 13 | 23 | Y |
| A. K. Dwivedi et al, 2019 | 10.1109/ACCESS.2018.2889437 | 1 | 11 | 117 | N |
| Millán CA et al, 2020 | 10.3390/ijerph17020498 | 1 | 11 | 16 | N |
| Decharatanachart P et al, 2021 | 10.1186/s12876-020-01585-5 | 1 | 13 | 80 | Y |
| Grothen AE et al, 2020 | 10.1200/CCI.20.00101 | 1 | 2 | 36 | N |
| Musulin J et al, 2021 | 10.3390/ijerph18084287 | 2 | 1 | 127 | N |
| R. A. Rahman et al, 2020 | 10.1109/ACCESS.2020.3029154 | 1 | 6 | 22 | N |
| Lee Y et al, 2018 | 10.1016/j.jad.2018.08.073 | 1 | 6 | 26 | Y |
| Mak KK et al, 2019 | 10.1016/j.psychres.2019.03.001 | 3 | 6 | 17 | N |
| Charalambides M. et al, 2020 | 10.12968/hmed.2019.0322 | 1 | 2 | 9 | N |
| Bernert RA et al, 2020 | 10.3390/ijerph17165929 | 1 | 6 | 87 | N |
| Chee ML et al, 2021 | 10.3390/ijerph18094749 | 2 | 1 | 14 | N |
| Barua I et al, 2021 | 10.1055/a-1201-7165 | 1 | 2 | 5 | Y |
| Raffort J et al, 2020 | 10.1016/j.jvs.2019.12.026 | 1 | 11 | 34 | N |
| Adamidi ES et al, 2021 | 10.1016/j.csbj.2021.05.010 | 2 | 1 | 101 | N |
| Jin P et al, 2020 | 10.1007/s00432-020-03304-9 | 1 | 2 | 64 | N |
| Kocak B et al, 2020 | 10.2214/AJR.20.22847 | 1 | 2 | 30 | N |
| Abd-Alrazaq A. et al, 2020 | 10.2196/20756 | 2 | 1 | 82 | N |
| Jones OT et al, 2021 | 10.2196/23483 | 1 | 2 | 16 | N |
| Murray NM et al, 2020 | 10.1136/neurintsurg-2019-015135 | 1 | 8 | 20 | N |
| Shen J et al, 2019 | 10.2196/10010 | 3 | 24 | 9 | N |
| Scardoni A et al, 2020 | 10.1016/j.jiph.2020.06.006 | 2 | 1 | 27 | N |
| Geng X et al, 2020 | 10.1016/bs.pmbts.2020.04.015 | 1 | 6 | 60 | N |
| Marka A et al, 2019 | 10.1186/s12880-019-0307-7 | 1 | 2 | 39 | N |
| Kumar H et al, 2019 | 10.1016/j.cmpb.2019.07.002 | 1 | 13 | 19 | Y |
| Valente IR et al, 2016 | 10.1016/j.cmpb.2015.10.006 | 1 | 12 | 22 | N |
| R. M. Sarmento et al, 2020 | 10.1109/RBME.2019.2934500 | 1 | 8 | 103 | N |
| Balakrishnan R et al, 2021 | 10.1016/j.compmedimag.2021.101867 | 1 | 8 | 37 | N |
| Dallora AL et al, 2019 | 10.1371/journal.pone.0220242 | 1 | 20 | 26 | Y |
| Hassanipour S et al, 2019 | 10.1016/j.injury.2019.01.007 | 1 | 15 | 10 | Y |
| Sufriyana H et al, 2020 | 10.2196/16503 | 1 | 18 | 142 | Y |
| Burlacu A et al, 2021 | 10.3390/medicina57060538 | 1 | 11 | 16 | N |
| Bang CS et al, 2021 | 10.1016/j.gie.2020.11.025 | 1 | 2 | 21 | Y |
| Ferrante di Ruffano L et al, 2018 | 10.1002/14651858.CD013186 | 1 | 2 | 42 | N |
| Nikolaou V et al, 2020 | 10.1016/j.rmed.2020.106093 | 1 | 12 | 14 | N |
| Kedra J et al, 2019 | 10.1136/rmdopen-2019-001004 | 1 | 15 | 55 | N |
| Islam MM et al, 2020 | 10.1016/j.cmpb.2020.105320 | 1 | 9 | 23 | Y |
| Anteby R et al, 2021 |  | 1 | 15 | 16 | N |
| Soffer S et al, 2020 | 10.1016/j.gie.2020.04.039 | 1 | 13 | 19 | Y |
| Ghaderzadeh M et al, 2021 | 10.1155/2021/6677314 | 2 | 1 | 37 | N |
| Ebrahimighahnavieh MA et al, 2020 | 10.1016/j.cmpb.2019.105242 | 1 | 6 | 114 | N |
| Nielsen KB et al, 2019 | 10.1016/j.oret.2018.10.014 | 1 | 9 | 11 | N |
| Prados-Privado M et al, 2020 | 10.3390/jcm9113579 | 1 | 15 | 12 | N |
| R. Fernandes et al, 2020 | 10.22489/CinC.2020.333 | 1 | 11 | 28 | N |
| Nindrea RD et al, 2018 | 10.22034/APJCP.2018.19.7.1747 | 1 | 2 | 11 | Y |
| Bruin W et al, 2019 | 10.1016/j.pnpbp.2018.08.005 | 1 | 6 | 12 | N |
| Groot OQ et al, 2020 | 10.1097/CORR.0000000000001360 | 1 | 15 | 12 | N |
| Mehta TI et al, 2020 | 10.1016/j.jvir.2020.08.002 | 1 | 2 | 14 | Y |
| Medic G et al, 2019 | 10.12688/f1000research.20498.2 | 1 | 24 | 20 | N |
| Payrovnaziri SN et al, 2020 | 10.1093/jamia/ocaa053 | 3 | 24 | 42 | N |
| Ford E et al, 2016 | 10.1093/jamia/ocv180 | 3 | 24 | 67 | N |
| Sugano D et al, 2020 | 10.1097/MOU.0000000000000822 | 1 | 2 | 40 | Y |
| Sanfelici R et al, 2020 | 10.1016/j.biopsych.2020.02.009 | 1 | 6 | 44 | Y |
| W. Khan et al, 2021 | 10.1109/ACCESS.2021.3069937 | 1 | 12 | 65 | N |
| Cresswell K et al, 2020 | 10.1177/1460458219900452 | 3 | 24 | 5 | N |
| Patil S et al, 2019 | 10.1111/jop.12854 | 1 | 2 | 7 | N |
| Dallora AL et al, 2017 | 10.1371/journal.pone.0179804 | 1 | 6 | 37 | N |
| Le Glaz A et al, 2021 | 10.2196/15708 | 1 | 6 | 58 | N |
| Senders JT et al, 2018 | 10.1016/j.wneu.2017.09.149 | 1 | 8 | 30 | N |
| Xie C.-Y. et al, 2021 | 10.3390/cancers13102469 | 1 | 2 | 26 | N |
| V. Soares de Siqueira et al, 2020 | 10.1109/COMPSAC48688.2020.0-215 | 1 | 11 | 45 | N |
| T. B. Lacerda et al, 2020 | 10.23919/CISTI49556.2020.9140861 | 1 | 2 | 32 | N |
| Vallmuur K. et al, 2015 | 10.1016/j.aap.2015.03.018 | 1 | 22 | 16 | N |
| Mekki A et al, 2019 | 10.1016/j.ejca.2019.06.020 | 1 | 2 | 65 | N |
| Bracher-Smith M et al, 2021 | 10.1038/s41380-020-0825-2 | 1 | 6 | 13 | N |
| Fleuren LM et al, 2020 | 10.1007/s00134-019-05872-y | 1 | 1 | 28 | Y |
| Liu NT et al, 2015 | 10.1016/j.burns.2015.07.001 | 1 | 14 | 15 | N |
| Shatte ABR et al, 2019 | 10.1017/S0033291719000151 | 1 | 6 | 300 | N |
| Alabi RO et al, 2021 | 10.1016/j.artmed.2021.102060 | 1 | 2 | 41 | N |
| Senanayake S et al, 2019 | 10.1016/j.ijmedinf.2019.103957 | 1 | 16 | 18 | N |
| J. Tulloch et al, 2020 | 10.1109/ACCESS.2020.3035327 | 1 | 5 | 37 | Y |
| Yassin NIR et al, 2018 | 10.1016/j.cmpb.2017.12.012 | 1 | 2 | 154 | N |
| Rjoob K et al, 2020 | 10.1016/j.jelectrocard.2020.08.013 | 1 | 11 | 14 | Y |
| Shung D et al, 2019 | 10.1007/s10620-019-05645-z | 1 | 13 | 14 | N |
| Kareemi H et al, 2021 | 10.1111/acem.14190 | 2 | 24 | 23 | N |
| Oskar S. et al, 2020 | 10.1007/s40572-020-00282-5 | 3 | 24 | 42 | N |
| Muralitharan S et al, 2021 | 10.2196/25187 | 1 | 20 | 24 | N |
| Levman J et al, 2015 | 10.1016/j.nicl.2015.09.017 | 1 | 6 | 50 | N |
| Smith A et al, 2017 | 10.1016/j.semarthrit.2017.06.005 | 1 | 15 | 18 | N |
| Pons E et al, 2016 | 10.1148/radiol.16142770 | 1 | X | 67 | N |
| Koleck TA et al, 2019 | 10.1093/jamia/ocy173 | 3 | 24 | 14 | N |
| Kreimeyer K et al, 2017 | 10.1016/j.jbi.2017.07.012 | 3 | 24 | 86 | N |
| Wang S et al, 2020 | 10.1530/EJE-19-0968 | 1 | 9 | 24 | Y |
| Bradley A et al, 2019 | 10.1097/MPA.0000000000001312 | 1 | 2 | 6 | N |
| A. Baldominos et al, 2020 | 10.1109/ACCESS.2020.2973006 | 2 | 1 | 47 | N |
| Payedimarri AB et al, 2021 | 10.3390/ijerph18094499 | 2 | 1 | 8 | N |
| Mahajan SM et al, 2018 | 10.1177/1474515118799059 | 1 | 11 | 334 | Y |
| Gautam R et al, 2020 | 10.1007/s10916-019-1519-7 | 1 | 8 | 136 | Y |
| Xu L et al, 2021 | 10.1007/s00415-021-10508-7 | 1 | 8 | 28 | N |
| Gregório T et al, 2018 | 10.1186/s12874-018-0613-8 | 1 | 3 | 59 | Y |
| Lai Q et al, 2020 | 10.3748/wjg.v26.i42.6679 | 1 | 2 | 9 | N |
| Wongkoblap A et al, 2017 | 10.2196/jmir.7215 | 1 | 6 | 48 | N |
| Albahri AS et al, 2020 | 10.1007/s10916-020-01582-x | 2 | 1 | 8 | N |
| Singh D et al, 2020 | 10.1016/j.cmpb.2019.105074 | 1 | 2 | 18 | N |
| Brinker TJ et al, 2018 | 10.2196/11936 | 1 | 2 | 13 | N |
| Antosik-Wójcińska AZ et al, 2020 | 10.1016/j.ijmedinf.2020.104131 | 1 | 6 | 27 | N |
| Albahri OS et al, 2020 | 10.1016/j.jiph.2020.06.028 | 2 | 1 | 11 | N |
| M. A. Rashidan et al, 2021 | 10.1109/ACCESS.2021.3060753 | 1 | 6 | 20 | N |
| Milne-Ives M et al, 2020 | 10.2196/20346 | 3 | 24 | 31 | N |
| Aziz M et al, 2020 | 10.1111/jgh.15070 | 1 | 13 | 3 | Y |
| M. O. Khairandish et al, 2020 | 10.1109/ICOIACT50329.2020.9332131 | 1 | 2 | 8 | N |
| Hung K et al, 2020 | 10.1259/dmfr.20190107 | 1 | 15 | 50 | N |
| V. Nunavath et al, 2019 | 10.1109/ICT-DM47966.2019.9032935 | 3 | 24 | 100 | N |
| Burke TA et al, 2019 | 10.1016/j.jad.2018.11.073 | 1 | 6 | 35 | N |
| Li J et al, 2020 | 10.11152/mu-2402 | 1 | 2 | 10 | Y |
| Layeghian Javan S et al, 2018 | 10.1016/j.jbi.2018.10.008 | 1 | 11 | 75 | N |
| Silva K et al, 2020 | 10.1016/j.ijmedinf.2020.104268 | 1 | 5 | 23 | Y |
| Shillan D et al, 2019 | 10.1186/s13054-019-2564-9 | 3 | 24 | 258 | N |
| Wingfield LR et al, 2020 | 10.1002/lt.25772 | 1 | 13 | 9 | N |
| Li WT et al, 2020 | 10.1186/s12911-020-01266-z | 2 | 1 | 151 | Y |
| Miles J et al, 2020 | 10.1186/s41512-020-00084-1 | 2 | 24 | 25 | Y |
| Al-Garadi MA et al, 2016 | 10.1016/j.jbi.2016.05.005 | 2 | 1 | 20 | N |
| Langerhuizen DWG et al, 2019 | 10.1097/CORR.0000000000000848 | 1 | 15 | 10 | N |

Table 1. Systematic reviews included in the analysis

# Appendix III

| **Author year** | **Health/Disease** | **Data type** | **Ouctome** | **Modelling technique: R,ML,DL,RL** | **Challenges** | **Opportunities** |
| --- | --- | --- | --- | --- | --- | --- |
| Salod Z et al, 2020 | Breast cancer | Boold analysis dataset | Prediction | ML | Few published evidence. Review protocols should inlcude other type of ML paradigms, such as semi-supervised. The small sample size was the main challenge. | The use of ML for Breast Cancer screening and detection is promising |
| Fleuren LM et al, 2020 | Sepsis | Vital signs, demographic data, lab data, arterial blood gas and observations. | Prediction | ML | Systematic reporting and clinical implementation studies are needed to bridge the gap between bytes and bedside. | Individual machine learning models can accurately predict sepsis onset ahead of time. Although they present alternatives to traditional scoring systems, between-study heterogeneity limits the assessment of pooled results. |
| Kothari G et al, 2021 | Non-small cell lung cancer | Radiomics (CT and PET) | Pronostic | R, ML | Radiomics based models for lung cancer have to date demonstrated modest prognostic capabilities | The literature must use standardised radiomics features, a robust feature selection and deep learning techniques to improve imaging-based models. |
| Yuan M et al, 2019 | Public health surveillance | EHR | Detection | R, ML | These approaches have yet to be adopted widely in practice | Aberration detection methods are used routinely in public health practice and they continue to be a focus of research. Evidence is acumulating that newer methods, such as ML methods, can out perform traditional time-series methods |
| M. A. S. A. Husaini et al, 2020 | Breast cancer | Medical images (thermal images) | Classification | DL | Small sample size used in the experiments, the heavy dependence on specific databases, image enhancement techniques applied and the limited number of advanced deep learning models. | Validity and potential of using theromography for early breast cancer detection by capturing the differences in thermal distribution across the breast tissue. ANN and deep learning models could achieve an acceptable level of detection accuracy but they can be improved. |
| Bellinger C et al, 2017 | Air pollution epidemiology | Environmental data | Forecast or predict | ML, DL | New data from sensors and storage mediums that enable larger, better quality data is needed to have fruitfull applications. | Data mining related to temporal and geo-spacial mining and deep learning is increasingly being applied in air pollution epidemiology. |
| Kearns WR et al, 2019 | Health dialog systems | Conversational agents | Screening | ML | Dialog systems have been widely applied to health care, most studies are not reproducible making direct comparison. | The adoption of standard evaluation and reporting methods for health dialog systems is required to demonstrate clinical significance |
| N. F. Zulkifli et al, 2020 | Addiction | Not available | Prediction and classification | ML, DL | Research studies on ML application in addiction psychiatry is quite limited and not much research published regarding using ML in predicting treatment success | Overview on the effectiveness of ML method used in addiction studies and in hope to benefits researcher/scientist or even the public to be aware of the potential of applying ML in addiction studies |
| Wang W et al, 2020 | Stroke | EHR, cohort and clinical trial data | Predicting outcomes (such as mortality and functional outcome) | ML | Few publications met basic reporting standards for clinical prediction tools and none made their models available in a way which could be used or evaluated. Major improvements in ML study conduct and reporting are needed before it can meaningfully be considered for practice. | The use of ML for predicting stroke outcomes is increasing |
| Gupta V et al, 2020 | Hematopoietic Stem Cell Transplantation (HSCT) | EHR, hospital data and registries. Clinical data (most used). Also genomic and biological data, imaging data and others. |  | ML | The evidence is not suciently robust to determine the optimal ML technique to use in the clinical setting and/or what minimal data variables are required | A wide range of ML techniques are being increasingly applied |
| Dreisbach C et al, 2019 | Pain, fatigue and sleep disturbance. And others | EHR and ePAT (electronic patient-authored text) | Identification, detection, extraction, and/or description of symptom terms | ML | Future research should consider the needs of patients expressed through electronic forms and its relevance to symptom science. | Understanding the role that electronic communications plays in health communication an real-time assessment of symptoms, through the use of NLP and text mining, is critical to a pacient-centered health system |
| Gregório T et al, 2018 | Intracerebral hemorrhage | Patients | Prediction | R, ML | Logistic regression based risk scores are particularly promising given their good performance and ease of application | Prognostic tools for ICH discriminated well for mortality and functional outcome in derivation studies but methodological issues require confirmation of these findings in validation studies |
| Zhong J et al, 2021 | Osteosarcoma | Radiomics | Treatment response prediction and survival prediction | ML, DL | Improvements in study design, validation, and open science needs to be made to demonstrate the generalizability of findings and to achieve clinical applications. Widespread application of RQS, pre-trained RQS scoring procedure, and modification of RQS in response to clinical needs are necessary | The overall scientific quality of included studies is insufficient; however, radiomics remains a promising technology for predicting treatment response, which might guide therapeutic decision-making and related to prognosis. |
| Karmegam D et al, 2020 | Psychological Surveillance | Social media Data | Screening | ML | Social media data do not represent the entire population. The Twitter API service allows users to collect only a sample of tweets and so the data are not the representative of the entire Twitter activity. There are chances on the no connection between the disaster location and the person who tweets. | Futher research needs to be conducted to use social media in disaster management |
| Harris M et al, 2019 | Pulmonary tuberculosis | Medical image (chest x-rays) | Diagnosis | ML, DL | more clinical studies are needed that minimize sources of potential bias to ensure validity of the findings outside of the study setting | We conclude that CAD programs are promising, but the majority of work thus far has been on development rather than clinical evaluation |
| M. N. Islam et al, 2020 | COVID-19 | Medical image and non-image data | Classification, forecasting and prediction | ML, DL | Supportive information, such as new datasets with high-dimensional features, more classes, and cooperation with the medical community are needed to add value to handling this health threat using AI and ML | The research has been classified into five different categories based on their objectives. AI and ML can be helpful in differentiating between seasonal flu and COVID-19 infections with acceptable accuracy and could guide governments and communities in the early control of the impact of the virus. |
| Lui TKL et al, 2020 | Gastrointestinal Endoscopy | Medical image | Detection of GI lesions | ML, DL | Most studies were based on retrospective reviews of selected images, which requires further validation in pro-spective trials | AI is accurate in the detection of upper GI neoplastic lesions and HP infection status |
| A. K. Dwivedi et al, 2019 | Heart Sounds | Registries (cardiac signal) | Segmentation and classification | ML | There are no robust methods for identification and classification of various events in the cardiac cycle. | A cost-effective system with precise automatic analysis of heart sounds may assist in early diagnosis and to improve the outcomes of cardiovascular diseases, furthermore with the combination of wearable mobile technologies |
| Millán CA et al, 2020 | Atrial Fibrillation | Registries (Photoplethysmographic signals) | Classification | ML, R | A lack of investigation and implementation of features in frequency domain and time-frequency domain was evident | The vast availability of pubilc data bases allow the development and comparison of AI models |
| Decharatanachart P et al, 2021 | Chronic Liver Diseases | Medical Image and clinical and laboratory data | Diagnosis of liver diseases | ML, DL | The validation is a crucial step to be warranted before implementing these AI-assisted systems in clinical practice | AI-assisted systems have promising potential for the diagnosis of liver fbrosis and NAFLD. |
| Grothen AE et al, 2020 | Cancer | EHR (pharmacy data) | Supporting treatment and clinical decision making (most common) | ML (25%), NLP (67%) and combined ML and NLP (8%) | Data sources and representations are often missing, challenging study replicability. There is no consistent format for reporting results, and one of the preferred metrics, F-score, is often missing. There is a resultant need for greater transparency of original data sources and performance of AI methods with pharmacy data to improve the translation of there results into meaningful outcomes. | This review demostrates that the application of AI data methods for pharmacy informatics and cancer epidemiology research is expanding. |
| Musulin J et al, 2021 | COVID-19 | Not available | Prediction | R, ML | While there are many high-quality items pre-published, and the early availability of such results is crucial for the situations as an ongoing pandemic, researchers should be careful when utilizing pre-published research and use their best judgment in determining the quality of published and peer-reviewed research of similar topic | AI-based algorithms have a clear application in COVID-19 epidemiological spread modeling and may be a crucial tool in the combat against coming pandemics |
| R. A. Rahman et al, 2020 | Mental Health Detection | Social media data | Detection | ML, DL | OSNs can provide complementary data, and the combination of the two approaches in detecting mental health can improve future research | OSN exhibit high potential as data sources of mental health problems detection, and its use must be explored in addition to traditional mental health detection methods based on face-to-face interiews, self-reporting, or questionaire distribution. |
| Lee Y et al, 2018 | Depression | Neuroimaging EEG or MRI), phonomenolofical, genetic or combined data. | Prediction | R, ML | An integrative approach may more effectively model neurobiolo-gical components as functional modules of pathophysiology embedded within the complex, social dynamics thatinfluence the phenomenology of mental disorders | Machine learning algorithms provide a powerful conceptual and analytic framework capable ofintegrating multiple data types and sources |
| Mak KK et al, 2019 | Addiction | Clinical data, demographic data, social media data | Detection | R, ML, RL: supervised learning (13), unsupervised learning (2) and reinforcement learning (2) | Further studies of the potential applications of machine learning methods in precision psychiatry and neuroscience are needed | These results suggest that machine learning methods, particularly supervised learning are increasingly used in addiction psychiatry for providing evidence for medical decision. |
| Charalambides M. et al, 2020 | Melanoma | Medical images | Detection | DL | A likely future scenario may involve artificial intelligence systems with rapid analytic skills serving as support tools to physicians of all levels of training and experience, supplementing and augmenting the diagnosis of challenging lesions | Use of artificial intelligence remains largely experimental in dermatology. Maintaining the doctor–patient relationship is central to the provision of holistic medical care and the dermatologist’s flexibility, adaptability and experience cannot be substituted. |
| Bernert RA et al, 2020 | Suicidal behaviour | EMR , epidomiologic surveys or social media user data. | Prediction of suicidal behaviour | ML | AI may crucially inform the early detection of suicide risk, triage, and treatment development, with important methodological and statistical cautions. The application of NLP to social media in particular, and integration of AI with real-time suicide risk assessments, holds unique promise to impact the prevention of suicide on a broad scale. | There are newly-identified risk variables or approaches such as point to sleep, circadian, and neural substrates, and NLP-derived indices of speech or user data that could be used |
| Chee ML et al, 2021 | COVID-19 | ICU and ED patients | Diagnostic and pronostic | ML, DL | Our findings underscore the need for improvements to facilitate safe and effective clinical adoption of AI applications, for and beyond the COVID-19 pandemic. | Current AI applications for COVID-19 are not ready for deployment in acute care settings, given their limited scope and poor quality. Available reporting methodologies should be used. |
| Barua I et al, 2021 | Polyp and colorectal cancer | Clinical data | Detection | DL , ML | AI tools should focus on increased detection of advanced adenomas, where the potential for prevention without may be largest | Our review shows that AI-based polyp detection systems increase the mean number of polyps detected per colonoscopy and mean number of adenomas detected per colonoscopy, but shows no difference when it comes to advanced adenomas. |
| Raffort J et al, 2020 | Abdominal aortic aneurysm | Medical image | Segmentation and prediction | ML | AI-driven data management may lead to the development of computational programs for the prediction of aneurysm evolution and risk of rupture as well as postoperative outcomes. These tools have not been used in clinical settings yet. | AI could be used to help surgeons in preoperative planning, representing an attractive tool for decision-making and may facilitate development of personalized therapeutic approaches for patients. |
| Adamidi ES et al, 2021 | COVID-19 | Medical image, demographic data and clinica data. | Prognostic (50), diagnostic (28) and screening (14) | R, ML, DL | Novel technologies such as explainable AI and Federated Learningcould prove to be critical in tackling volatile crises like the COVID-19 pandemic | AI methods are critical tools for utilizing therapidly growing body of COVID-19 positive patient datasets, with avast contribution in the fight against this pandemic. |
| Jin P et al, 2020 | Gastric cancer | Genomic data, Medical image (endoscopic) | Diagnosis and treatment | ML, DL | The future of integrating AI technologies into clinical practice is promising. To facilitate this, multidisciplinary collaboration is mandatory. Furthermore, large randomized controlled trials are warranted to validate the AI models. | AI can be further investigated for the entire process of cancer management. In particular, some possible felds include early detection, pathology recognition, risk evaluation, treatment guidance, and outcome predictions |
| Kocak B et al, 2020 | Renal Cell carcinoma | Radiomics (CT and MRI) | Prediction | ML, DL | To bring AI-based renal mass characterization from research to practice, future studies need to improve modeling and performance evaluation strategies and pay attention to clinical utility and transparency issues. | The quality items were favorable for modeling and performance evaluation categories for most studies |
| Abd-Alrazaq A. et al, 2020 | COVID-19 | Radiology images, biological data, epidemiological data, clinical data, laboratory data, demographic data, guidelines, and news articles. | Diagnosis | DL (60), ML (29) and NLP(3) | Many of the proposed methods are not yet clinically accepted. Thus, the most rewarding research will be on methods promising value beyond COVID-19. More efforts are needed for developing standardized reporting protocols or guidelines for studies on AI | AI has the potential to fight against COVID-19 |
| Jones OT et al, 2021 | Cancer | EHR | Diagnosis | R, ML | Further evidence is needed on their performance using primary care data, implementation barriers, and cost-effectiveness before widespread adoption into routine primary care clinical practice can be recommended. | AI techniques have been applied to EHR-type data to facilitate early diagnosis of cancer, but their use in primary care settings is still at an early stage of maturity |
| Murray NM et al, 2020 | Ischemic stroke and large vessel occlusions | Medical imagen (CT and MRI) | Prediction and diagnosis | ML, DL | Standardization of performance assessment is needed in future studies. | AI may improve detection and rapid triage necessary for expedited treatment |
| Shen J et al, 2019 | Disease diagnosis | Not available | Diagnosis | ML, DL | Further studies should be extended to other types of medical imaging such as magnetic resonance imaging and other medical practices unrelated to images. | Current AI development has a diagnostic performance that is comparable with medical experts, especially in image recognition-related fields |
| Scardoni A et al, 2020 | Healthcare-associated infections (HAIs) | Not available | Screening | ML, R | While their adoption and impact for research, healthcare quality improvement, ornational surveillance purposes is still far from being explored | Available evidence mainly focuses on the development and testing of HAIs detection andprediction models |
| Geng X et al, 2020 | Autism Spectrum Disorder (ASD) | Medical image (17) and behavioral data (43) | Classification and prediction | R, ML, DL | The translation to clinical practicerequires extensive further research including external validation with large samplesize and optimized feature selection. | The use of multimodal features, e.g., combination of neuroimaging and behavior, it is worth further investigation to improve theprediction accuracy. |
| Marka A et al, 2019 | Nonmelanoma skin cancer | Medical Image | Detection | ML, R | Clinical studies are needed to assess whether these technologies can feasibly be implemented as a real-time aid for clinical diagnosis of NMSC | Most studies of image-based classifiers report performance greater than or equal to the reported diagnostic accuracy of the average dermatologist, but relatively few studies have presented a high level of evidence |
| Kumar H et al, 2019 | Pancreas segmentation | Medical Image (CT) | Segmentation | DL | The above findings indicate that the automation of pan- creas segmentation represents a considerable challenge as the performance of current automated pancreas segmentation algorithms is suboptimal. | Adopting standardised reporting on performance of pancreas segmentation algorithms and encouraging the use of benchmark pancreas segmentation datasets will allow future algorithms to be tested and compared more easily and fairly |
| Valente IR et al, 2016 | Pulmonary nodule | Medical image (CT) | Classification | ML, R | Further research is needed to develop current techniques and that new algorithm sare needed to overcome the identified drawbacks | The results of the review can be particularly useful for researchers working in the development and/or improvement of CADe systems for pulmonary nodule detection |
| R. M. Sarmento et al, 2020 | Stroke | Medical image (CT, MRI) | Classification | ML, DL | The issues that were addressed the most were the need to optimize computational techniques, the extraction and selection of more suitable hyperparameters and the limitations of hardware performance | The use of DIP and AI techniques in CAD systems has shown promising results, making them a fundamental tool to aid diagnoses and medical follow-ups |
| Balakrishnan R et al, 2021 | White matter hyperintensities (WMH) | Medical Image (MRI) | Segmentation | ML, DL | Data and code availability, bias in study design and ground truth generation influence the wider validation and applicability of these methods in clinical research | We found no evidence that favours deep learning methods over the more established k-NN, linear regression and unsupervised methods in this task. |
| J. Tulloch et al, 2020 | Diabetic Foot Ulcers | Medical image | Segmentation and classification | ML | Future research should address the following: direct comparison of ML applications with current standards of care, health economic analyses and large scale data collection. | ML offers a way in which the care of DFU patients can be substantially improved; from large scale data analysis, to at-home image analysis for personalised treatment and management |
| Hassanipour S et al, 2019 | Trauma patients | Not available | Prediction | ML, R | ANN model provides more accurately predictions for examining health-related outcomes such as death rate, survival rate and duration ofhospitalization in the intensive care unit. Other methods are found suboptimal | Using an ANN to predict thefinal implications of trauma patients can provide more accurate clinical decisions |
| Silva K et al, 2020 | Type 2 diabetes | Patients | Prediction | R, ML | Improvements to methodology, reporting and validation are needed before they can be used at scale. | We found evidence of good performance of ML models for T2DM prediction in the community. |
| Burlacu A et al, 2021 | Cardiovascular complications in Chronic Kidney Disease | Registries | Prediction | ML, R | There is a clear need for more extensive application of rigorous methodologies. Following the future prospective, randomized clinical trials, and thorough external validations, computational solutions will fill the gap in cardiovascular predictive tools for chronic kidney disease | The identified studies represent palpable trends in areas of clinical promise with an encouraging present-day performance |
| Bang CS et al, 2021 | Esophageal cancer | Medical image | Diagnosis | ML, DL | Lack of external validation and clinical applications should be overcome | CAD algorithms showed high accuracy for the automatic endoscopic diagnosis of esophageal cancer and neoplasms. |
| Ferrante di Ruffano L et al, 2018 | Skin Cancer | Medical Image | Diagnosis | ML | The evidence base is currently too poor to understand whether CAD system outputs translate to different clinical decision–making in practice. Insufficient data are available on the use of CAD in community settings, or for the detection of keratinocyte cancers. The evidence base for individual systems is too limited to draw conclusions on which might be preferred for practice. | In highly selected patient populations all CAD types demonstrate high sensitivity, and could prove useful as a back‐up for specialist diagnosis to assist in minimising the risk of missing melanomas |
| Nikolaou V et al, 2020 | Chronic Obstructive Pulmonary Disease (COPD) | EHR, genetic data and clinical data | Classification | ML | We believe that future research should be tasked with further investigating COPD phenotype(s) whose characteristics have not yet been fully explored. | This article reviewed research published in the last decade on COPD phenotypes identified using cluster analysis and validated with clini-cally meaningful outcomes. |
| Kedra J et al, 2019 | Rheumatic and musculoskeletal diseases (RMDs) | Not available | Not available | ML | Big data sources and types are varied within the field of RMDs, and methods used to analyse big data were heterogeneous. | These findings provide a current status, which will inform a European League Against Rheumatism taskforce on big data in RMDs. |
| Islam MM et al, 2020 | Diabetic retinopathy | Medical image (retinal fundus photographs) | Diagnosis | DL |  | The findings of our study showed that DL algorithms had high sensitivity and specificity for detecting referable DR from retinal fundus photographs. |
| Anteby R et al, 2021 | Liver fibrosis | Medical images | Classification | DL | There is a still limited number of retrospective studies. Clinicians should facilitate the use of this technology by sharing databases and standarized reports. This may optimize the noninvasive evaluation of liver fibrosis on a large scale | Deep learning has the potential to play an emerging role in liver fibrosis classification |
| Soffer S et al, 2020 | Wireless Capsule Endoscopy (WCE) | Medical images (WCE image) | Diagnosis | DL | Future prospective, multicenter studies are necessary for this technology to be implemented in the clinical use of WCE. Current research is based on retrospective studies with a high risk of bias. | Deep learning has achieved excellent performance for the detection of a range of diseases in WCE. |
| Ghaderzadeh M et al, 2021 | COVID-19 | Medical images (X-ray, CT) | Detection and diagnosis | DL | Developers should be careful to avoid overfitting and to maximize the generalizability and usefulness of COVID-19 DL diagnostic models. These models must be trained on large, heterogeneous datasets to cover all the available data space. | The application of deep learning in the field of COVID-19 radiologic image processing reduces false-positive and negative errors in the detection and diagnosis of this disease and offers a unique opportunity to provide fast, cheap, and safe diagnostic services to patients |
| Ebrahimighahnavieh MA et al, 2020 | Alzheimer's disease | Medical image (MRI, PET) | Detect | DL | Regardless of the final AD detection system, overfitting issues related to the dataset still need to be resolved. | Deep learning has enabled the development of AD detection systems. It is clear that combining these neuroimaging modalities can aid AD detection and can be used with other factors like memory test scores and genetic infor- mation to deliver a more accurate diagnosis. |
| Nielsen KB et al, 2019 | Diabetic retinopathy | Medical image | Prediction | DL | Challenges which may hinder such an implementation particularly revolves around ethical concerns regarding lack of trust in the diagnostic accuracy of computers | Advantages of implementing deep learning-based algorithms in DR-screening include reduction in manpower, cost of screening and issues relating to intra- and intergrader variability. |
| Prados-Privado M et al, 2020 | Dental Caries Diagnosis | Medical image | Classification and segmentation | ML | All this variability complicates the conclusions that can be made about the reliability or not of a neural network to detect and diagnose caries. A comparison between neural network and dentist results is also necessary | Each study included in this review used a different neural network and different outcome metrics. |
| R. Fernandes et al, 2020 | Cardiac arrhythmias | Registries | Classification | ML | As few authors use the same evaluation scheme for testing, it is difficult to make a fair comparison between methods. | Classification of cardiac arrhythmias through ECG is of increasing interest from the research groups, and ML classification is showing rising levels of performance. It would benefit both patients and clinicians. |
| Nindrea RD et al, 2018 | Breast cancer | Medical image (digital mammograms) and others | Diagnosis | ML | The proposed methodology does not determine the onset of breast cancer, which can be performed through mammographic diagnosis. | SVM algorithm is able to calculate breast cancer risk with better accuracy value than other machine learning algorithms. AI can encourage potential breast cancer-prone women to go the hospital for diagnostic tests. Therefore, the early diagnosis of breast cancer will be more effective, and the mortality rate of breast cancer will decrease |
| Bruin W et al, 2019 | Obsessive-compulsive disorder | Medical Image (MRI) | Diagnosis | ML | Although studies have shown promising results, sample sizes used are typically small which can lead to high variance of the estimated model accuracy, cohort-specific solutions and lack of generalizability of findings. Collaborative efforts to share both data and code allow for more extensive model assessment and will help the field advance to clinical applicability. Features important for classification were different across imaging modalities and widespread throughout the brain | . Studies providing an accurate benchmark for a series of well-known machine learning algorithms are valuable as they can provide insight into the diagnostic value of predictive models |
| Groot OQ et al, 2020 | Musculoskeletal Radiological Studies | Medical Image | Detection and classification | ML | Future studies should emphasize how ML models can complement clinicians, instead of analyzing the potential superiority of one versus the other. | ML models could improve the safety and effectiveness of patient care while working in conjunction with human counterparts. |
| Mehta TI et al, 2020 | Painful Osseous Metastases | Clinical data | Treatment | ML | The results of this meta-analysis indicate that RF ablation results in rapid, long-lasting, significant relief of osseous metastatic pain, but prospective, ideally randomized trials are necessary to fully elucidate its effect | RF ablation may represent an efficacious palliative treatment for painful, osseous metastatic disease. In particular, RF ablation may have specific applications for analgesic-resistant osseous metastatic pain, possibly more effective in axial over appendicular tumors |
| Liu NT et al, 2015 | Burn care | Clinical data and wound images | Diagnosis and prediction | ML | The impact of ML applications in clinical practice would be much enhanced by a common framework for reporting on the individual features, training criteria, and performances of algorithms as well as the way in which they were developed and the specifics of the environment in which they were deployed. | This review supported thepotential efficacy of ML in burn care/research |
| Payrovnaziri SN et al, 2020 | Analyse electronic health record data | EHR | Prediction | DL, ML (XAI) | Based on our review, we found that XAI evaluation in medicine has not been adequately and formally practiced. | Reproducibility remains a critical concern. Ample opportunities exist to advance XAI research in medicine. |
| Ford E et al, 2016 | Improve case detection | EMR | Detection | ML | More harmonization of reporting within EMR studies is needed, particularly standardized reporting of algorithm accuracy metrics like positive predictive value (precision) and sensitivity (recall) | Text in EMRs is accessible, especially with open source information extraction algorithms, and significantly improves case detection when combined with codes. |
| Sugano D et al, 2020 | Prostate cancer | Radiomics | Detection | ML | With further implementation and large-scale validation, radiomics has the potential to change the landscape of PCa detection, necessitating further prospective randomized investigation. | Radiomics is a promising new field, allowing for high-throughput analysis of imaging features for Pca detection. |
| Sanfelici R et al, 2020 | Psychosis | Medical image , clinical data and others | Diagnosis | R, ML | The review result on a substantial clinical and methodological heterogeneity currently affecting several aspects of the CHR field and limiting the clinical implementability of the proposed models | Future studies are warranted to investigate whether harmonizing procedures within precision psy-chiatry will lead to more reliable and reproducible translational research in the field. |
| W. Khan et al, 2021 | Pneumonia | Medical image (X-ray) | Prediction | ML, DL | The majority of the existing works have only used frontal radiographs. To improve the detection accuracy, lateral view radiographs should also be considered. The majority of work has been done on normal human lung anatomy. Few authors have incorporated nonimage features. The majority of the ML-based pneumonia identification systems lack transparency on how the data split was performed. Several models for pneumonia detection can perform up to 99% accuracy, however, often the sensitivity or specificity of the models are not promising | Traditional ML methods can be employed when high computational resources are scarce but cannot be employed for industrial-scale intelligent detection of pneumonia. Therefore, DL can be used. |
| Cresswell K et al, 2020 | Health and social care | Clinical data | Not available | ML | While the applied review methodology may be viewed as immature in an area where robust empirical evidence is nascent, it has helped to identify how the existing empirical evidence base should be conceptualised in this important but emerging field going forward. | The evidence of effectiveness of AI to support decision-making in health and social care settings is limited and contradictory, perhaps reflecting the variability in contexts in which technologies are deployed |
| Patil S et al, 2019 | Head and neck cancer | Genomic data | Prediction | ML | Use of larger multidimensional and heterogeneous data, application of more accurate validation results, and use of different techniques for classification and feature selection may pave the way forward for its use in clinical setting | We have found that ML techniques for the analysis of genomic data can play a role in the prognostic prediction of HNC |
| Dallora AL et al, 2017 | Dementia | Neuroimaging, cognitibe measures, genetic, lab test, demopraphic | Prediction | ML | Only two studies focused on prediction regarding populations, and those were the only two studies that applied MS techniques. We identified only a limited number of datasets are being used in the studies. | Most of the studies were concerned about predicting the development of AD in individuals with MCI using one of ML techniques. Neuroimaging data was the most common data to be fed into ML techniques |
| Le Glaz A et al, 2021 | Mental Health | EHR and social media | Classification | ML | Some language-specific features can improve the performance of NLP methods, and their extension to other languages should be more closely investigated. | Machine learning and NLP models have been highly topical issues in medicine in recent years and may be considered a new paradigm in medical research |
| Senders JT et al, 2018 | Neurosurgical (epilepsy, brain tumor, spinal lesions, neurovascular disease, movement disorders, traumatic brain injury, and hydrocephalus) | Clinical, neuropsychological, EEG, intracranial-EEG, MRI, proton emission tomography and pathology | Prediction | ML | Future studies should explorethe hurdles associated with the creation, validation, and deployment of ML modelsin clinical care parallel to the developmentof these methods, as well as ethical andsocietal implications of their adoption | ML models have great potential for improving neurosurgical outcome prediction. They can be a valuable aid for phy-sicians, patients, and their families in theprocess of surgical and medical decision-making. |
| Xie C.-Y. et al, 2021 | Esophageal cancer | Radiomics | Classification | R, ML, DL | Recent advances and future perspectives of the ML technique demonstrate its potential to provide novel quantitative imaging markers in medical imaging. Prospective large multi-center studies are suggested to improve the generalizability of ML techniques with standardized imaging protocols and harmonization between different centers | Patients stratified by ML models in different risk groups have a significant or borderline significant difference in survival outcomes. |
| V. Soares de Siqueira et al, 2020 | Heart diseases | ECHO (echocardiogram) images or videos | Classification | ML | The researches on this topic still demand optimized software that can be used in real-time. | DL methods may be the key to a successful automatization of the echocardiogram processes |
| T. B. Lacerda et al, 2020 | Cancer | Demographic, Clinical and Histopathological data | Prediction | ML | The results show gaps in current research as no studies were identified on ML using Comprehensive Geriatric Assessment (CGA), a fundamental tool that is used to improve caring for elderly patients with cancer. |  |
| Senanayake S et al, 2019 | Graft failure after kidney transplantation | Living and deceased donor transplants data (10). Deceased donor transplant (6). Living donor transplant information (1) | Prediciton | ML | Future research should focus on modelling time-to-event information using machine learning methods such as Survival tree , Random survival forest and Survival Support Vector Machine. | Based on reported gains in predictive performance, machine learning has the potential to improve kidney transplant outcome prediction and aid medical decision making |
| Mekki A et al, 2019 | Pituitary metastasis | Medical image (MR, PET/CT) | Diagnosis | ML | It has been possible to only evaluate the imaging of acute phase HP, when the problem of differentiating from PM is at its greatest | An image-based signature was developed with machine learning and validated fordifferentiating PM from HP. This tool could beused by clinicians for enhanced decision-making in cancer patients undergoing ICB treatment with new-onset, concerning lesions ofthe pituitary gland |
| Bracher-Smith M et al, 2021 | Psychiatric disorders | Genetic data | Prediction | ML | Key steps in model development and validation were frequently not performed or unreported. Comparison of discrimination across studies was constrained by heterogeneity of predictors, outcome and measurement, in addition to sample overlap within and across studies. Given widespread high risk of bias and the small number of studies identified, it is important to ensure established analysis methods are adopted. | Performance measures consequently demonstrated a wide range of abilities to discriminate between cases and controls |
| Sufriyana H et al, 2020 | Pregnancy Care | Registries | Prediction | R, ML | We recommend a reanalysis of existing LR models for several pregnancy outcomes by comparing them with those algorithms that apply standard guidelines | Prediction models with the best performances across studies were not necessarily those that used LR but also used random forest and gradient boosting that also performed well |
| Dallora AL et al, 2019 | Bone Age | Medical Image (Xray) | Diagnose -Classification (Bone age of a subject) | R,ML | Studies that considered ethnical differences were scarce and socioeconomic aspects were inexistent. high heterogeneity in the studies what makes the comparing them a challenge. | Studies considering socioeconomic differences, studies considering more ROIs other than hand and wrist. |
| Shatte ABR et al, 2019 | Mental health | Imaging data (n = 102), survey data (n=40), mobile and wearable sensor data (n=29) and social media data (n=28) | Detection and diagnosis (n = 190); Prognosis, treatment and support (n = 67); Public health applications (n = 26); Research and clinical administration (n = 17). | ML, R | The majority of studies identified focusing on the detection and diagnosis of mental health conditions, it is evident that there is significant room for the application of ML to other areas of psychology and mental health. | ML tools are becoming more accessible for researchers and clinicians, it is expected that the field will continue to grow and that novel applications for mental health will follow |
| Alabi RO et al, 2021 | Oral squamous cell carcinoma | Clinopathologic, imaging or genomic data. | Diagnosis | ML, DL | Lack of explainability, interpretability, and externally validation for generalizability prevents safely integrated into daily clinical practices. Also, regulatory frameworks for the adoption of these models in clinical practices are necessary. | Machine learning models have been reported to show promising performances for diagnostic and prognostic analyses in studies of oral cancer |
| Muralitharan S et al, 2021 | Clinical deterioration | Vital signs | Prediction | R, ML | There is a need for standardized outcome measures to allow for rigorous evaluation of performance across models. Further research needs to address the interpretability of model outputs by clinicians, clinical efficacy of these systems through prospective study design, and their potential impact in different clinical settings. | In studies that compared performance, reported results suggest that machine learning–based early warning systems can achieve greater accuracy than aggregate-weighted early warning systems but several areas for further research were identified |
| Vallmuur K. et al, 2015 | Injury surveillance | Textual injury data | Screening | ML | There were limitations to the machine learning techniques: (1) problems with generalizability of results, (2) source data issues, (3) complex model application challenges, (4) limitations in the integration of domain and data mining knowledge. | With advances in data mining techniques, increased capacity for analysis of large databases, and involvement of computer scientists in the injury prevention field, along with more comprehensive use and description of quality assurance methods in text mining approaches, it is likely that we will see a continued growth and advancement in knowledge of text mining in the injury field |
| Yassin NIR et al, 2018 | Breast cancer | Medical Image (DM, digital mammogram; US, ultrasound; MRI, magnetic resonance imaging; Microscopic images; and IRT, infrared thermography) | Classification | ML, R | The few availability of standardized public image databases that contain images from different image modalities for the same case compromises the dependency of more than one image modality in classification task and combine information from multiple views. | SVM classifier has been used extensively for breast tissue classification purposes. Deep learning classifier is a promising trend that appeared in the recent years. |
| Rjoob K et al, 2020 | Recording ECGs | Registies (ECG) | Prediction | ML | These findings highlight the importance of developing new algorithms such as deep learning to detectelectrode misplacement and interchange errors with a high sensitivity and specificity, especially to detect LA-LL interchange | Ourfindings highlight opportunities for enhancing ECG data quality and clinical decision makingthrough the accurate detection of lead misplacement. |
| Shung D et al, 2019 | Acute Gastrointestinal Bleeding | Laboratory values, clinical variables and demographic data | Prediction | ML, R | Limitations include heterogeneity of ML models, inconsistent comparisons of ML models with clinical risk scores, and high risk of bias | ML generally provided good–excellent prognostic performance in patients with GIB, and artifcial neural networks tended to outperform other ML models. ML was better than clinical risk scores for mortality in upper GIB |
| Kareemi H et al, 2021 | Emergency Department | Triage vital signals an labotatory test | Prediction | ML, R | Future research should investigate the clinical impacts of machine learning models in interventional trials. | Machine learning models appear to have better diagnostic and prognostic prediction performance compared to usual care for ED patients with a variety of presentations |
| Oskar S. et al, 2020 | Early-life environmental exposures | Environmental data | Prediction | ML | In order to conduct high-quality research, we need to ensure that the ML techniques we use are appropriate for the study questions and are implemented and reported in a way that can critically be assessed by the scientific community | As the amount of complex environmental data continues to grow, we anticipate greater use of ML to address challenges that cannot be easily handled with traditional analytic methods |
| Young IJB et al, 2019 | Incident reporting and adverse event analysis | IRS (Incident Reporting System), EHR, and others | Classification | ML | NLP enables insights to be drawn from larger datasets it may improve the learning from adverse events in healthcare | NLP can generate meaningful information from unstructured data in the specific domain of the classification of incident reports and adverse events. Understanding what or why incidents are occurring isimportant in adverse event analysis |
| Levman J et al, 2015 | Neurodevelopmental disorders (autism, attention-deficil hyperactivity disorder, epilepsy and neuropsychiatric disorders) | Medical image (pediatric MRI) | Classification | ML, R | There is room for improving MVA techniques and adapting them to better characterize, diagnose and detect neurological developmental disorders in pre-adult populations | Multivariate analysis technologies can play a useful role in helping to answer questions about structural and functional organization and development in the brain. |
| Smith A et al, 2017 | Musculoskeletal pain | Medical Image (MRI and fMRI) | Classification | ML | The combination of various behavioural, genotype and phenotype data into analyses to assist with development of sensitive and specific signatures | There is preliminary and emerging evidence that MVPA analyses of structural or functional MRI are able to discriminate between patients and healthy controls, and also discriminate betweennoxious and non-noxious stimulation |
| Pons E et al, 2016 | Radiology | EHR and others | Diagnosis | ML | Performance of NLP systems is generally high, but not many applications are actually being used in routine clinical practice or research. Proliferation of NLP applications in radiology may improve by establishing performance requirements, report standardization, and external validation. | Through automation, NLP applications can process large amounts of data and bring new functionality to clinical workflows |
| Koleck TA et al, 2019 | Symptoms in EHR | EHR | Predicting | ML | Future NLP studies should concentrate on the investigation of symptoms and symptom documentation in EHR free-text narratives. Efforts should be undertaken to examine patient characteristics and make symptom-related NLP algorithms or pipelines and vocabularies openly available. | The current focus of this field is on the development of methods to extract symptom information and the use of symptom information for disease classification tasks rather than on the investigation of symptoms themselves. |
| Kreimeyer K et al, 2017 | Capture clinical information | Clinical notes, radiology reports, pathology reports, biomedical literature | Capturing and standarizing unstructures clinical information | ML |  | The information collected and evaluated here will be important for prioritizing development of new approaches for clinical NLP |
| Wang S et al, 2020 | Diabetic retinopathy | Medical Image and EHR | Screening | DL | To further improve diagnostic accuracy of neural networks, researchers might need to develop new algorithms rather than simply enlarge sample sizes of training sets or optimize image quality. | State-of-the-art neural networks could effectively detect clini cal significant DR |
| Bradley A et al, 2019 | Pancreatic cancer | Registries | Treatment and prediction | ML, R | The future direction of research relies on expanding out view of the multidisciplinary team to include professionals from computing and data science backgroungs with algorithms developed in conjuction with clinicians and viewed as aids, not replacement, to traditional clinical decision-making | Using this expanse of data to facilitate decision-making in a meaningful way for individual patients is beyond the capabilities of the human mind working in isolation. It is in this context thatmachine learning holds the greatest potential by being able to handle large amounts of data and integrate large, complex, and varied databases |
| A. Baldominos et al, 2020 | Infections | Demographisc, environmental data, EHR, clinical data, laboratory test, etc… | Prediction | R, ML | Modelswith different features and algorithms used for infection predicion should be evaluated over time, to detect technological trends and advances in the discipline. | Automatic diagnosis of infectious diseases using computational intelligence is well documented in the medical literature. |
| Payedimarri AB et al, 2021 | COVID-19 | Empirical and simulation data | Prediction | R, ML | New COVID-19 variants could have different transmission patterns and the national vaccination program will substantially change the effects of interventions over time | We concluded that AI and ML could be of help for policy makers to define the strategies for containing the COVID-19 pandemic. |
| Mahajan SM et al, 2018 | Heart failure | Patients | Screening | R, ML | More work is needed for calibration, external validation, and deployment of such models for clinical use | Large volumes of diverse electronic data and new statistical methods have improved the predictive power of the models over the past two decades |
| Gautam R et al, 2020 | Neurological disorders | Medical Image | Diagnosis | DL | There is a potential challenge on exploring the performance of Restricted Boltzmann Machine, Deep Boltzmann Machine and Deep Belief Network for diagnosis of different human neuropsychiatric and neurological disorders. | The research breaches are identified and it is witnessed that there is more scope in the diagnosis of migraine, cerebral palsy and stroke using different deep learning models. |
| Xu L et al, 2021 | Amyotrophic lateral sclerosis | Patients | Prediction | ML, R | More models based on low- or middle-income countries should be established. Furthermore, the availability of predictors, appropriate model presentation as well as standard modelling process should be emphasized to help practitioners, policy makers, and guideline developers decide which model to use. | A number of ALS prognostic models from developed countries have been developed, but several methodological pitfalls in the model development were observed. |
| Medic G et al, 2019 | Critical care | EHR, vital signs, registries | Prediction | ML | Large datasets are required for training these algorithms; making it imperative to appropriately address, challenges such as class imbalance, correct labelling of data and missing data. Recommendations are formulated for the development and successful adoption of CDS systems. | Increasing use of Machine Learning for CDS in all three areas |
| Lai Q et al, 2020 | Hepatocellular cancer | Patients | Prediction | DL, ML | Improved transferability and reproducibility will facilitate the widespread use of AI methodologies | AI applied to survival prediction after HCC treatment provided enhanced accuracy compared with conventional linear systems of analysis |
| Wongkoblap A et al, 2017 | Mental Health Disorders | Social media | Classify and predict | ML, R | Assembling large, high-quality datasets of social media users with mental disorder is problematic, not only due to biases associated with the collection methods, but also with regard to managing consent and selecting appropriate analytics techniques | Despite an increasing number of studies investigating mental health issues using social network data, some common problems persist. |
| Albahri AS et al, 2020 | COVID-19 | Patients | Detecting and diagnosing | ML | Findings showed that researchers must proceed with insights they gain, focus on identifying solutions for CoV problems, and introduce new improvements | The growing emphasis on data mining and ML techniques in medical fields can provide the right environment for change and improvement |
| Singh D et al, 2020 | Breast cancer | Image Thermography | Classification | ML | Existing intelligent systems also need improvement in commercially feasible interface with physicians, to validate the diagnosis | The advent of computer-aided diagnostics in healthcare field has proved to be very effective in improving the role of ther- mography in detection of breast cancer |
| Brinker TJ et al, 2018 | Skin Cancer | Medical Image | Classification of dermatoscopic patterns | DL | It is very difficult and impossible to compare the performance of published classification results since many authors use nonpublic datasets for training and/or testing. Future publications should use publicly available benchmarks and fully disclose methods used for training to allow comparability. | CNNs display a high performance as state-of-the-art skin lesion classifiers. |
| Antosik-Wójcińska AZ et al, 2020 | Bipolar disorder | Apps | Prediction | ML | Smartphone-based diagnostic methods are relatively new, therefore many monitoring apps have some shortcomings. | The management of BD could be significantly improved by monitoring of illness activity via smartphone |
| Albahri OS et al, 2020 | COVID-19 | Medical Images (CT, X-ray) | Detection and classification | ML, DL | The challenges of such gap are discussed, and the process of evaluation and benchmarking of COVID-19 AI classificationtechniques is considered a multi-complex attribute problem. | Evaluating and benchmarking of AI classification techniques (i.e. binary, multi-class, multi-labelled andhierarchical classifications), which could be used in the detectionand diagnosis of COVID-19 medical image, is a critical gap of relatedliterature. |
| M. A. Rashidan et al, 2021 | Autism Spectrum Disorder (ASD) | Registies | Screening and classifying | ML | In terms of methodology, several primary studies suggested to have larger training dataset in order to overcome the overfitting problem. | It can be assumed that the emotion recognition with assisted by technology, for evaluating and classifying affective states could help to improve efficacy in therapy sessions between therapists and individuals with ASD |
| Milne-Ives M et al, 2020 | Conversational Agents | Not available | Prediction | ML | The quality of many of the studies was limited, and improved study design and reporting are necessary to more accurately evaluate the usefulness of the agents in health care and identify key areas for improvement. Further research should also analyze the cost-effectiveness, privacy, and security of the agents | The studies generally reported positive or mixed evidence for the effectiveness, usability, and satisfactoriness of the conversational agents investigated, but qualitative user perceptions were more mixed |
| Aziz M et al, 2020 | Colonoscopy | Registies | Treatment | DL |  |  |
| M. O. Khairandish et al, 2020 | Brain tumor | Medical Image (MRI, CT) | Diagnosis | ML | The confidence in the research results in term of accuracy for the detection of brain tumors still needs to be increased. | Building a software applications can be very useful to solve real cases. |
| Hung K et al, 2020 | Dental and maxillofacial radiology | Medical Image (X-ray) | Screening | ML | It is still necessary to further verify the reliability and applicability of the AI models prior to transferring these models into clinical practice | The AI models proposed in the studies included exhibited wide clinical applications in DMFR |
| V. Nunavath et al, 2019 | Disaster Management | Social Networks (predominantly) | Classification (predominantly) | AI, ML, DL | As a future work, we would like to explore the potential of AI, ML and DL are for both natural and human-made disaster management. | Most of the articles for natural disasters focus on classification, prediction, best safest routes for evacuation, sentiment analysis, detection, and building decision support systems. |
| Burke TA et al, 2019 | Suicidal behaviour | EHR | Prediction | ML | Low paper sample size, inconsistent reporting procedures resulting in an inability to compare model accuracy across studies, and lack of model validation on external samples. | Leveraging machine learning techniques to further predictive accuracy and identify novel indicators will aid in the prediction and prevention of suicide |
| Li J et al, 2020 | Breast cancer | Registies | Classification (discriminating benign and malignant breast masses) | DL | There is a basis of evidence for further investigation on the S-Detect technique | S-Detect exhibited a favourable diagnostic value in assisting physicians discriminating benign and malignant breast masses and it can be considered as a useful complement for conventional US |
| Layeghian Javan S et al, 2018 | Cardiac arrest | Registies | Prediction and Classify | R, ML | Future research should be carried out to evaluate the efficiency of rarely-used algorithms and to address the challenges of external validation, implementation and adoption of machine learning models in real clinical environments. | According to the results, machine learning techniques can improve the outcome of cardiac arrest prediction |
| Arji G et al, 2019 | Traditional medicine | Not available | Classification (most common) | ML | It is suggested more studies be performed by means of other techniques such as complex network analysis, text mining and genetic algorithms in this domain. In addition to, test and validation of machine learning methods in a clinical setting are necessary, thus, a collaboration of researchers and traditional medicine spe- cialists to provide suitable models is recommended. | Syndrome differentiation was the main application domain of machine learning methods in traditional medicine. Among DM methods, BNS, ANNs, SVM and decision trees were identified as most popular methods for knowledge discovery in TM |
| Shillan D et al, 2019 | Intensive care units (ICU) | Patients | Prediction | ML | The sample sizes used in many published studies are too small to exploit the potential of these methods. Methodological and reporting guidelines are needed, particularly with regard to the choice of method and validation of predictions, to increase confidence in reported findings and aid in translating findings towards routine use in clinical practice. | The rate of publication of studies using machine learning to analyse routinely collected ICU data is increasing rapidly. |
| Wingfield LR et al, 2020 | Liver Transplantation | Patients (deceased donor liver transplantation) | Prediction | ML | The high accuracy of AI may come at a cost of losing explainability (to patients and clinicians) on how the technology works. | AI techniques can provide high accuracy in predicting graft survival based on donors and recipient variables. When compared with the standard techniques, AI methods are dynamic and are able to be trained and validated within every population. |
| Li WT et al, 2020 | COVID-19 | Influenza patients | Classification and diagnose | ML |  | Computational methods trained on large clinical datasets could yield ever more accurate COVID-19 diagnostic models to mitigate the impact of lack of testing. |
| Miles J et al, 2020 | Triage acuity of patient at Emergency Care System (ECS) | Patients who require a process of triage at ECS | Prediction | ML, R | There was no clear benefit of using one technique over another; however, models derived by logistic regression were more transparent in reporting model performance. Future studies should adhere to reporting guidelines and use these at the protocol design stage. | Machine-learning methods appear accurate in triaging undifferentiated patients entering the Emergency Care System. |
| Al-Garadi MA et al, 2016 | Pandemic | Social Networks | Classification | ML, R | An OSN-based surveillance system requires comprehensive adoption, enhanced geographical identification system, and advanced algorithms and computational linguistics to eliminate its limitations and challenges. OSN is probably to never replace traditional surveillance, but it can offer complementary data that can work best when integrated withtraditional data. | OSN data contain significant information that can be used to track a pandemic. Different from traditional surveys and clinical reports, in which the data collection process is time consuming atcostly rates, OSN data can be collected almost in real time at a cheaper cost. Additionally, the geograph-ical and temporal information can provide exploratory analysis of spatiotemporal dynamics of infectiousdisease spread. |
| Langerhuizen DWG et al, 2019 | Fracture | Medical Image (Xray, CT) | Detection and classification | ML | Inadequate reference standard assignments to train and test AI is the biggest hurdle before integration into clinical workflow. The next step will be to apply AI to more challenging diagnostic and therapeutic scenarios when there is absence of certitude. Future studies should also seek to address legal regulation and better determine feasibility of implementation in clinical practice. | Preliminary experience with fracture detection and classification using AI shows promising performance. AI may enhance processing and communicating probabilistic tasks in medicine, including orthopaedic surgery. |

# Appendix IV

Complete results of the data analysis.

| **Universal Healthcare Coverage** | | | | |
| --- | --- | --- | --- | --- |
|  | | | |  |
| **Categorical variables** | | | | **n (%)** |
| **Data Source** | | | |  |
|  | Public and private | | | 67 (68,37) |
|  | Public | | | 28 (28,57) |
|  | Not available | | | 3 (3,06) |
| **Data type** | | | |  |
|  | Medical Image | | | 38 (38,78) |
|  | Clinical data | | | 15 (15,31) |
|  | Registries | | | 11 (11,22) |
|  | Electronic health record (EHR) | | | 10 (10,20) |
|  | Mixed types of data | | | 8 (8,16) |
|  | Radiomics | | | 5 (5,10) |
|  | Social media data | | | 4 (4,08) |
|  | Genomic data | | | 3 (3,06) |
|  | Not available | | | 4 (4,08) |
| **Predictors** | | | |  |
|  | Regions of interest | | | 39 (39,8) |
|  | Not available | | | 23 (23,47) |
|  | Mixed and others | | | 15 (15,33) |
|  | Clinical or demographic variables | | | 6 (6,12) |
|  | Signal features and vital signs | | | 6 (6,12) |
|  | Text (keyword) | | | 5 (5,10) |
|  | Histological and texture features | | | 4 (4,08) |
| **Outcome** | | | |  |
|  | Prediction/Screening | | | 32 (32,65) |
|  | Detection | | | 24 (24,49) |
|  | Classification | | | 18 (18,37) |
|  | Others | | | 6 (6,12) |
|  | Treatment | | | 4 (4,08) |
|  | Segmentation | | | 4 (4,08) |
|  | Prediction and Classification |  |  | 4 (4,08) |
|  | Detection and Classification | | | 4 (4,08) |
|  | Not available | | | 2 (2,04) |
| **Type of AI** | | | |  |
|  | Machine learning | | | 44 (44,90) |
|  | Regression and Machine learning | | | 23 (23,47) |
|  | Machine and deep learning | | | 16 (16,33) |
|  | Deep learning | | | 13 (13,27) |
|  | Regression, machine and deep learning | | | 2 (2,04) |
| **Predominant technique** | | | |  |
|  | Support vector machine (SVM) | | | 24 (24,49) |
|  | Convolutional neural network (CNN) | | | 20 (20,41) |
|  | Others | | | 15 (15,31) |
|  | Not available | | | 13 (13,27) |
|  | Artificial neural network (ANN) | | | 11 (11,22) |
|  | Random forest (RF) | | | 6 (6,12) |
|  | Regression (R) | | | 5 (5,10) |
|  | Logistic regression (LR) | | | 4 (4,08) |
| **Validation** | | | |  |
|  | Not available | | | 51 (52,04) |
|  | Internal | | | 32 (32,65) |
|  | Internal and external | | | 12 (12,24) |
|  | External | | | 5 (5,10) |
| **Predominant indicator** | | | |  |
|  | Accuracy | | | 31 (31,63) |
|  | Mixed | | | 24 (24,49) |
|  | Area Under Curve (AUC) | | | 20 (20,41) |
|  | Sensitivity and specificity | | | 12 (12,24) |
|  | Not available | | | 7 (7,14) |
|  | C statistics | | | 4 (4,08) |
| **Risk of bias assessment** | | | |  |
|  | No | | | 60 (61,22) |
|  | Yes | | | 38 (38,78) |
|  |  | QUADAS-2 | | 17 (17,35) |
|  |  | Others | | 7 (7,14) |
|  |  | PROBAST | | 4 (4,08) |
|  |  | Cochrane | | 3 (3,06) |
|  |  | ROB | | 2 (2,04) |
|  |  | Egger's and Begg's | | 2 (2,04) |
| **Cuantitative variables** | | | |  |
| Number of Systematic Reviews analyzed | | | | 98 (100) |
| Number of Meta-analysis analyzed | | | | 27 (27,55) |
| Number of included reviews | | | | 40,57±50,53 |
| Number of compared reviews | | | | 27,81±26,87 |
| AMSTAR Score | | | | 4,05±1,99 |

Table 2. Complete descriptive results for UHC

| **Health Emergencies Protection** | | | | |
| --- | --- | --- | --- | --- |
|  | | | |  |
| **Categorical variables** | | | | **n (%)** |
| **Data Source** | | | |  |
|  | Public and private | | | 12 (75) |
|  | Public | | | 4 (25) |
| **Data type** | | | |  |
|  | Clinical data | | | 4 (25) |
|  | Medical image | | | 3 (18,75) |
|  | Mixed types of data | | | 3 (18,75) |
|  | Social media data | | | 2 (12,5) |
|  | Not available | | | 2 (12,5) |
|  | Laboratory test | | | 1 (6,25) |
|  | Empirical data |  |  | 1 (6,25) |
| **Predictors** | | | |  |
|  | Not available | | | 6 (37,5) |
|  | Regions of interest | | | 4 (25) |
|  | Text (keyword) | | | 2 (12,5) |
|  | Clinical or demographic variables | | | 2 (12,5) |
|  | Genome sequences | | | 1 (6,25) |
|  | Mixed | | | 1 (6,25) |
| **Outcome** | | | |  |
|  | Prediction/Screening | | | 8 (50) |
|  | Detection | | | 4 (25) |
|  | Classification | | | 4 (25) |
| **Type of AI** | | | |  |
|  | Regression and Machine learning | | | 7 (43,75) |
|  | Machine and deep learning | | | 4 (25) |
|  | Machine learning | | | 3 (18,75) |
|  | Deep learning | | | 1 (6,25) |
|  | Regression, machine and deep learning | | | 1 (6,25) |
| **Predominant technique** | | | |  |
|  | Convolutional neural network (CNN) | | | 3 (18,75) |
|  | Others | | | 3 (18,75) |
|  | Not available | | | 3 (18,75) |
|  | Logistic regression (LR) | | | 2 (12,5) |
|  | Artificial neural network (ANN) | | | 1 (6,25) |
|  | Natural language processing (NLP) | | | 1 (6,25) |
|  | Random forest (RF) | | | 1 (6,25) |
|  | Support vector machine (SVM) | | | 1 (6,25) |
|  | Decision tree | | | 1 (6,25) |
| **Validation** | | | |  |
|  | Not available | | | 9 (56,25) |
|  | Internal and external | | | 4 (25) |
|  | Internal | | | 3 (18,75) |
| **Predominant indicator** | | | |  |
|  | Accuracy | | | 4 (25) |
|  | Area under curve (AUC) | | | 4 (25) |
|  | Mixed | | | 2 (12,5) |
|  | Not available | | | 2 (12,5) |
|  | Sensitivity and specificity | | | 1 (6,25) |
|  | R² | | | 1 (6,25) |
|  | C statistics | | | 1 (6,25) |
| **Risk of bias assessment** | | | |  |
|  | No | | | 12 (75) |
|  | Yes | | | 4 (25) |
|  |  | PROBAST | | 3 (18,75) |
|  |  | ROB | | 1 (6,25) |
| **Cuantitative variables** | | | |  |
| Number of Systematic Reviews analyzed | | | | 16 (100) |
| Number of Meta-analysis analyzed | | | | 2 (12,5) |
| Number of included reviews | | | | 46,75±44,66 |
| Number of compared reviews | | | | 88,00±89,10 |
| AMSTAR Score | | | | 4,06±1,98 |

Table 3. Complete descriptive results for Health Emergencies

| **Better Health and Wellbeing** | | | |
| --- | --- | --- | --- |
|  | | |  |
| **Categorical variables** | | | **n (%)** |
| **Data Source** | | |  |
|  | Public and private | | 8 (53,3) |
|  | Public | | 6 (40) |
|  | Private | | 1 (6,7) |
| **Data type** | | |  |
|  | Electronic health record (EHR) | | 5 (33,3) |
|  | Clinical data | | 3 (20) |
|  | Not available | | 3 (20) |
|  | Environmental data | | 2 (13,3) |
|  | Social media data | | 1 (6,7) |
|  | Mixed types of data | | 1 (6,7) |
| **Predictors** | | |  |
|  | Not available | | 7 (46,7) |
|  | Text (keyword) | | 6 (40) |
|  | Clinical or demographic variables | | 1 (6,7) |
|  | Mixed | | 1 (6,7) |
| **Outcome** | | |  |
|  | Prediction/Screening | | 7 (46,7) |
|  | Detection | | 5 (33,3) |
|  | Classification | | 2 (13,3) |
|  | Not available | | 1 (6,7) |
| **Type of AI** | | |  |
|  | Machine learning | | 9 (60) |
|  | Machine and deep learning | | 4 (26,7) |
|  | Regression and machine learning | | 1 (6,7) |
|  | Regression, machine and reinforcement learning | | 1 (6,7) |
| **Predominant technique** | | |  |
|  | Natural language processing (NLP) | | 5 (33,3) |
|  | Support vector machine (SVM) | | 2 (13,3) |
|  | Neural network (NN) | | 2 (13,3) |
|  | Not available | | 2 (13,3) |
|  | Regression (R) | | 1 (6,7) |
|  | Artificial neural network (ANN) | | 1 (6,7) |
|  | Convolutional neural network (CNN) | | 1 (6,7) |
|  | Tree-based | | 1 (6,7) |
| **Validation** | | |  |
|  | Not available | | 10 (66,7) |
|  | Internal | | 4 (26,7) |
|  | Internal and external | | 1 (6,7) |
| **Predominant indicator** | | |  |
|  | Not available | | 8 (53,5) |
|  | Mixed | | 3 (20) |
|  | Accuracy | | 2 (13,3) |
|  | Area under curve (AUC) | | 1 (6,7) |
|  | Sensitivity and specificity | | 1 (6,7) |
| **Risk of bias assessment** | | |  |
|  | No | | 12 (80) |
|  | Yes | | 3 (20) |
|  |  | Cochrane | 2 (13,3) |
| **Cuantitative variables** | | |  |
| Number of Systematic Reviews analyzed | | | 15 (100) |
| Number of Meta-analysis analyzed | | | 0 (0) |
| Number of included reviews | | | 62,00±66,36 |
| AMSTAR Score | | | 3,67±1,54 |

Table 4. Complete descriptive results for Better Health and Wellbeing

# Appendix V

| **Author, year** | **1** | **2** | **3** | **4** | **5** | **6** | **7** | **8** | **9** | **10** | **11** | **AMSTAR**  **Score** | **Quality of**  **the review** |
| --- | --- | --- | --- | --- | --- | --- | --- | --- | --- | --- | --- | --- | --- |
| Salod Z et al, 2020 | N | Y | Y | N | N | Y | N | N | N | N | N | 3 | LOW |
| Arji G et al, 2019 | N | N | Y | N | N | N | Y | N | N | N | N | 2 | LOW |
| Kothari G et al, 2021 | Y | Y | Y | N | N | Y | Y | N | Y | N | N | 6 | MODERATE |
| Yuan M et al, 2019 | N | Y | Y | N | N | Y | Y | N | N | N | N | 4 | LOW |
| M. A. S. A. Husaini et al, 2020 | N | N | N | N | N | N | N | N | N | N | N | 0 | LOW |
| Bellinger C et al, 2017 | N | Y | Y | N | N | Y | N | N | N | N | N | 3 | LOW |
| Kearns WR et al, 2019 | N | Y | Y | N | N | Y | N | N | Y | N | N | 4 | LOW |
| N. F. Zulkifli et al, 2020 | N | Y | Y | N | N | Y | N | N | N | N | N | 3 | LOW |
| Wang W et al, 2020 | Y | Y | Y | N | N | Y | Y | Y | N | N | N | 6 | MODERATE |
| Gupta V et al, 2020 | N | Y | Y | N | N | Y | N | N | N | N | N | 3 | LOW |
| Dreisbach C et al, 2019 | N | Y | Y | N | N | Y | Y | N | N | N | N | 4 | LOW |
| Young IJB et al, 2019 | N | Y | Y | Y | N | Y | Y | Y | N | N | N | 6 | MODERATE |
| Zhong J et al, 2021 | Y | Y | Y | N | N | N | Y | Y | Y | Y | N | 7 | MODERATE |
| Karmegam D et al, 2020 | N | Y | Y | N | N | Y | Y | N | Y | N | N | 5 | MODERATE |
| Harris M et al, 2019 | Y | Y | Y | N | N | Y | Y | Y | Y | N | N | 7 | MODERATE |
| M. N. Islam et al, 2020 | N | Y | Y | N | N | Y | N | N | N | N | N | 3 | LOW |
| Lui TKL et al, 2020 | N | Y | Y | N | N | Y | Y | Y | Y | Y | N | 7 | MODERATE |
| A. K. Dwivedi et al, 2019 | N | Y | Y | N | N | Y | N | N | N | N | N | 3 | LOW |
| Millán CA et al, 2020 | N | N | Y | N | N | N | N | N | N | N | N | 1 | LOW |
| Decharatanachart P et al, 2021 | Y | Y | Y | N | N | Y | Y | N | Y | Y | N | 7 | MODERATE |
| Grothen AE et al, 2020 | N | Y | Y | N | N | N | N | N | N | N | Y | 3 | LOW |
| Musulin J et al, 2021 | N | Y | Y | N | N | N | N | N | N | N | N | 2 | LOW |
| R. A. Rahman et al, 2020 | N | Y | Y | N | N | Y | Y | Y | N | N | N | 5 | MODERATE |
| Lee Y et al, 2018 | N | Y | N | N | N | Y | Y | Y | Y | Y | N | 6 | MODERATE |
| Mak KK et al, 2019 | N | Y | Y | N | N | Y | N | N | N | N | N | 3 | LOW |
| Charalambides M. et al, 2020 | N | N | N | N | N | Y | N | N | N | N | N | 1 | LOW |
| Bernert RA et al, 2020 | N | Y | Y | N | N | Y | N | N | N | N | N | 3 | LOW |
| Chee ML et al, 2021 | N | Y | Y | N | N | Y | Y | Y | N | N | N | 5 | MODERATE |
| Barua I et al, 2021 | Y | Y | Y | Y | N | Y | Y | Y | Y | Y | N | 9 | ACCEPTABLE |
| Raffort J et al, 2020 | N | N | N | N | N | Y | N | N | N | N | N | 1 | LOW |
| Adamidi ES et al, 2021 | N | Y | Y | N | N | Y | Y | N | N | N | N | 4 | LOW |
| Jin P et al, 2020 | N | Y | Y | N | N | Y | N | N | N | N | N | 3 | LOW |
| Kocak B et al, 2020 | N | N | N | N | N | Y | Y | Y | Y | N | N | 4 | LOW |
| Abd-Alrazaq A. et al, 2020 | N | Y | Y | Y | N | N | N | N | N | N | N | 3 | LOW |
| Jones OT et al, 2021 | Y | Y | Y | N | Y | Y | Y | Y | Y | N | N | 8 | ACCEPTABLE |
| Murray NM et al, 2020 | N | Y | Y | N | N | Y | N | N | N | N | N | 3 | LOW |
| Shen J et al, 2019 | N | Y | Y | N | N | Y | Y | N | N | N | N | 4 | LOW |
| Scardoni A et al, 2020 | N | Y | Y | N | N | Y | Y | Y | Y | N | N | 6 | MODERATE |
| Geng X et al, 2020 | N | Y | Y | N | N | Y | N | N | N | N | N | 3 | LOW |
| Marka A et al, 2019 | Y | Y | Y | N | N | Y | Y | Y | N | N | N | 6 | MODERATE |
| Kumar H et al, 2019 | N | N | Y | N | N | Y | N | N | Y | N | N | 3 | LOW |
| Valente IR et al, 2016 | N | N | Y | N | N | Y | N | N | N | N | N | 2 | LOW |
| R. M. Sarmento et al, 2020 | N | N | Y | N | N | N | N | N | N | N | N | 1 | LOW |
| Balakrishnan R et al, 2021 | Y | Y | Y | N | N | Y | Y | Y | N | N | N | 6 | MODERATE |
| Dallora AL et al, 2019 | Y | Y | Y | N | N | Y | Y | Y | N | N | N | 6 | MODERATE |
| Hassanipour S et al, 2019 | N | Y | Y | N | N | Y | Y | N | Y | N | N | 5 | MODERATE |
| Sufriyana H et al, 2020 | Y | Y | Y | N | Y | N | Y | N | Y | N | N | 6 | MODERATE |
| Burlacu A et al, 2021 | N | Y | Y | N | N | Y | Y | Y | N | N | N | 5 | MODERATE |
| Bang CS et al, 2021 | Y | Y | Y | N | N | Y | Y | N | Y | Y | N | 7 | MODERATE |
| Ferrante di Ruffano L et al, 2018 | N | Y | Y | N | Y | Y | Y | N | Y | Y | Y | 8 | ACCEPTABLE |
| Nikolaou V et al, 2020 | N | Y | Y | N | Y | Y | N | N | N | N | N | 4 | LOW |
| Kedra J et al, 2019 | N | N | N | N | N | N | N | N | N | N | N | 0 | LOW |
| Islam MM et al, 2020 | N | Y | Y | N | N | Y | Y | N | Y | N | N | 5 | MODERATE |
| Anteby R et al, 2021 | Y | Y | Y | N | N | Y | Y | N | Y | N | N | 6 | MODERATE |
| Soffer S et al, 2020 | N | N | N | N | N | Y | Y | N | Y | N | N | 3 | LOW |
| Ghaderzadeh M et al, 2021 | N | Y | Y | N | N | Y | N | N | N | N | N | 3 | LOW |
| Ebrahimighahnavieh MA et al, 2020 | N | N | Y | N | N | N | Y | N | N | N | N | 2 | LOW |
| Nielsen KB et al, 2019 | N | Y | Y | N | N | Y | Y | N | N | N | N | 4 | LOW |
| Prados-Privado M et al, 2020 | N | Y | Y | N | N | Y | Y | N | N | N | N | 4 | LOW |
| R. Fernandes et al, 2020 | N | Y | Y | N | N | Y | N | N | N | N | N | 3 | LOW |
| Nindrea RD et al, 2018 | N | Y | Y | N | N | Y | Y | N | N | N | N | 4 | LOW |
| Bruin W et al, 2019 | N | Y | N | N | N | Y | N | N | N | N | N | 2 | LOW |
| Groot OQ et al, 2020 | N | Y | Y | N | N | Y | Y | Y | N | N | N | 5 | MODERATE |
| Mehta TI et al, 2020 | N | Y | Y | N | N | N | Y | Y | Y | Y | N | 6 | MODERATE |
| Medic G et al, 2019 | N | Y | Y | N | N | Y | N | N | N | N | N | 3 | LOW |
| Payrovnaziri SN et al, 2020 | N | Y | Y | N | N | Y | N | N | N | N | N | 3 | LOW |
| Ford E et al, 2016 | N | Y | Y | N | N | Y | N | N | N | N | N | 3 | LOW |
| Sugano D et al, 2020 | N | N | Y | N | N | Y | N | N | N | N | N | 2 | LOW |
| Sanfelici R et al, 2020 | N | N | Y | N | N | Y | N | N | Y | N | N | 3 | LOW |
| W. Khan et al, 2021 | N | N | Y | N | N | Y | Y | N | N | N | N | 3 | LOW |
| Cresswell K et al, 2020 | Y | Y | Y | Y | N | Y | Y | Y | Y | N | N | 8 | ACCEPTABLE |
| Patil S et al, 2019 | Y | Y | Y | Y | N | Y | Y | Y | N | N | N | 7 | MODERATE |
| Dallora AL et al, 2017 | N | Y | Y | N | N | N | Y | N | N | N | N | 3 | LOW |
| Le Glaz A et al, 2021 | Y | Y | Y | N | N | N | N | N | Y | N | N | 4 | LOW |
| Senders JT et al, 2018 | N | Y | Y | N | N | Y | N | N | N | N | N | 3 | LOW |
| Xie C.-Y. et al, 2021 | N | N | Y | N | N | Y | N | N | N | N | N | 2 | LOW |
| V. Soares de Siqueira et al, 2020 | N | Y | Y | N | N | N | N | N | N | N | N | 2 | LOW |
| T. B. Lacerda et al, 2020 | Y | N | Y | N | N | Y | Y | N | N | N | N | 4 | LOW |
| Vallmuur K. et al, 2015 | N | N | Y | N | N | Y | N | N | Y | N | N | 3 | LOW |
| Mekki A et al, 2019 | N | N | N | N | N | Y | N | N | N | N | N | 1 | LOW |
| Bracher-Smith M et al, 2021 | Y | Y | Y | N | N | Y | Y | Y | N | N | N | 6 | MODERATE |
| Fleuren LM et al, 2020 | Y | Y | Y | N | N | N | Y | N | Y | N | N | 5 | MODERATE |
| Liu NT et al, 2015 | N | N | Y | N | N | Y | N | N | N | N | N | 2 | LOW |
| Shatte ABR et al, 2019 | N | Y | Y | N | N | Y | N | N | N | N | N | 3 | LOW |
| Alabi RO et al, 2021 | N | Y | Y | N | N | Y | Y | Y | N | N | N | 5 | MODERATE |
| Senanayake S et al, 2019 | N | Y | Y | N | N | Y | Y | N | N | N | N | 4 | LOW |
| J. Tulloch et al, 2020 | N | Y | Y | N | N | Y | Y | N | N | N | N | 4 | LOW |
| Yassin NIR et al, 2018 | N | N | Y | N | N | Y | N | N | N | N | N | 2 | LOW |
| Rjoob K et al, 2020 | N | Y | Y | N | N | Y | Y | N | N | N | N | 4 | LOW |
| Shung D et al, 2019 | N | Y | Y | N | N | Y | Y | Y | N | N | N | 5 | MODERATE |
| Kareemi H et al, 2021 | Y | Y | Y | Y | N | Y | Y | N | Y | N | N | 7 | MODERATE |
| Oskar S. et al, 2020 | N | N | Y | N | N | Y | N | N | N | N | Y | 3 | LOW |
| Muralitharan S et al, 2021 | N | Y | Y | N | N | Y | N | N | N | N | Y | 4 | LOW |
| Levman J et al, 2015 | N | N | N | N | N | Y | N | N | N | N | N | 1 | LOW |
| Smith A et al, 2017 | Y | Y | Y | N | N | Y | Y | N | N | N | N | 5 | MODERATE |
| Pons E et al, 2016 | N | Y | Y | N | N | N | Y | N | N | N | Y | 4 | LOW |
| Koleck TA et al, 2019 | N | Y | Y | N | N | Y | N | N | N | N | N | 3 | LOW |
| Kreimeyer K et al, 2017 | N | Y | Y | N | N | Y | N | N | N | N | N | 3 | LOW |
| Wang S et al, 2020 | N | N | Y | N | N | N | Y | N | Y | N | N | 3 | LOW |
| Bradley A et al, 2019 | Y | Y | Y | N | N | Y | Y | Y | N | N | N | 6 | MODERATE |
| A. Baldominos et al, 2020 | N | Y | Y | N | N | Y | N | N | N | N | N | 3 | LOW |
| Payedimarri AB et al, 2021 | N | Y | Y | Y | N | N | N | N | N | N | N | 3 | LOW |
| Mahajan SM et al, 2018 | N | Y | Y | N | N | N | Y | N | N | N | N | 3 | LOW |
| Gautam R et al, 2020 | N | N | N | N | N | Y | N | N | N | N | N | 1 | LOW |
| Xu L et al, 2021 | N | Y | Y | N | N | N | Y | N | Y | N | N | 4 | LOW |
| Gregório T et al, 2018 | N | Y | Y | N | N | Y | Y | Y | Y | N | N | 6 | MODERATE |
| Lai Q et al, 2020 | N | Y | Y | N | N | Y | Y | N | N | N | N | 4 | LOW |
| Wongkoblap A et al, 2017 | N | Y | Y | N | N | Y | N | N | N | N | N | 3 | LOW |
| Albahri AS et al, 2020 | N | Y | Y | N | N | Y | N | N | N | N | N | 3 | LOW |
| Singh D et al, 2020 | N | N | Y | N | N | Y | N | N | N | N | N | 2 | LOW |
| Brinker TJ et al, 2018 | N | N | Y | N | N | Y | N | N | N | N | N | 2 | LOW |
| Antosik-Wójcińska AZ et al, 2020 | N | Y | Y | N | N | Y | N | N | N | N | N | 3 | LOW |
| Albahri OS et al, 2020 | N | Y | Y | N | N | N | N | N | N | N | N | 2 | LOW |
| M. A. Rashidan et al, 2021 | N | Y | Y | N | N | Y | Y | N | N | N | N | 4 | LOW |
| Milne-Ives M et al, 2020 | Y | Y | Y | N | N | Y | Y | N | N | N | Y | 6 | MODERATE |
| Aziz M et al, 2020 | N | Y | Y | N | N | Y | Y | N | Y | Y | N | 6 | MODERATE |
| M. O. Khairandish et al, 2020 | N | Y | N | N | N | Y | Y | N | N | N | N | 3 | LOW |
| Hung K et al, 2020 | Y | Y | Y | N | N | Y | Y | N | N | N | N | 5 | MODERATE |
| V. Nunavath et al, 2019 | N | Y | N | N | N | Y | N | N | N | N | N | 2 | LOW |
| Burke TA et al, 2019 | N | Y | Y | N | N | Y | N | N | N | N | N | 3 | LOW |
| Li J et al, 2020 | N | Y | Y | N | N | Y | Y | Y | Y | Y | N | 7 | MODERATE |
| Layeghian Javan S et al, 2018 | N | Y | Y | N | N | Y | N | N | N | N | N | 3 | LOW |
| Silva K et al, 2020 | Y | Y | Y | N | N | Y | Y | Y | Y | Y | N | 8 | ACCEPTABLE |
| Shillan D et al, 2019 | N | Y | Y | N | N | N | N | N | N | N | N | 2 | LOW |
| Wingfield LR et al, 2020 | Y | Y | Y | N | N | Y | Y | Y | Y | N | N | 7 | MODERATE |
| Li WT et al, 2020 | N | N | N | N | N | Y | N | N | Y | N | N | 2 | LOW |
| Miles J et al, 2020 | Y | Y | Y | Y | Y | Y | Y | Y | Y | N | N | 9 | ACCEPTABLE |
| Al-Garadi MA et al, 2016 | N | Y | Y | N | N | Y | Y | Y | N | N | N | 5 | MODERATE |
| Langerhuizen DWG et al, 2019 | Y | Y | Y | N | N | Y | Y | Y | N | N | Y | 7 | MODERATE |

Table 5. AMSTAR evaluation and individual score
